# A novel gain-of-function phosphorylation site modulates PTPN22 inhibition of TCR signaling

**DOI:** 10.1016/j.jbc.2024.107393

**Published:** 2024-05-21

**Authors:** Chuling Zhuang, Shen Yang, Carlos G. Gonzalez, Richard I. Ainsworth, Sheng Li, Masumi Takayama Kobayashi, Igor Wierzbicki, Leigh-Ana M. Rossitto, Yutao Wen, Wolfgang Peti, Stephanie M. Stanford, David J. Gonzalez, Ramachandran Murali, Eugenio Santelli, Nunzio Bottini

**Affiliations:** 1 Department of Medicine, Altman Clinical and Translational Research Institute, University of California, San Diego, California, USA; 2 Department of Medicine, Kao Autoimmunity Institute, Cedars-Sinai Medical Center, Los Angeles, California, USA; 3 Department of Pharmacology, University of California, San Diego, California, USA; 4 Skaggs School of Pharmacy and Pharmaceutical Sciences, University of California, San Diego, California, USA; 5 Department of Medicine, University of California, San Diego, California, USA; 6 Department of Molecular Biology and Biophysics, University of Connecticut Health, Farmington, Connecticut, USA; 7 Department of Biomedical Sciences, Cedars-Sinai Medical Center, Los Angeles, California, USA; 8 Samuel Oschin Comprehensive Cancer Institute, Cedars-Sinai Medical Center, Los Angeles, California, USA

**Keywords:** autoimmunity, phosphorylation, tyrosine phosphatase, catalysis, T cell receptor, transcriptomics, phosphoproteomics, PTPN22, allosteric regulation, intrinsically disordered region

## Abstract

Protein tyrosine phosphatase nonreceptor type 22 (PTPN22) is encoded by a major autoimmunity gene and is a known inhibitor of T cell receptor (TCR) signaling and drug target for cancer immunotherapy. However, little is known about PTPN22 posttranslational regulation. Here, we characterize a phosphorylation site at Ser^325^ situated C terminal to the catalytic domain of PTPN22 and its roles in altering protein function. In human T cells, Ser^325^ is phosphorylated by glycogen synthase kinase-3 (GSK3) following TCR stimulation, which promotes its TCR-inhibitory activity. Signaling through the major TCR-dependent pathway under PTPN22 control was enhanced by CRISPR/Cas9-mediated suppression of Ser^325^ phosphorylation and inhibited by mimicking it *via* glutamic acid substitution. Global phospho-mass spectrometry showed Ser^325^ phosphorylation state alters downstream transcriptional activity through enrichment of Swi3p, Rsc8p, and Moira domain binding proteins, and next-generation sequencing revealed it differentially regulates the expression of chemokines and T cell activation pathways. Moreover, *in vitro* kinetic data suggest the modulation of activity depends on a cellular context. Finally, we begin to address the structural and mechanistic basis for the influence of Ser^325^ phosphorylation on the protein’s properties by deuterium exchange mass spectrometry and NMR spectroscopy. In conclusion, this study explores the function of a novel phosphorylation site of PTPN22 that is involved in complex regulation of TCR signaling and provides details that might inform the future development of allosteric modulators of PTPN22.

Protein phosphorylation is an essential regulator of cellular function in response to extracellular stimuli by virtue of its ability to reversibly affect different aspects of protein function, from enzymatic activity to protein–protein interaction and the recruitment of effector molecules at the appropriate sites. Protein tyrosine phosphatases (PTPs) and kinases (PTKs) mediate multiple signal transduction pathways *via* their catalytic activities. In turn, phosphorylation-based modification of PTPs and PTKs themselves is a major regulatory process for controlling their function and ultimately the transduction of intracellular signaling pathways (1).

The hematopoietic-specific PTP nonreceptor type 22 (PTPN22) is a key PTP strongly associated with susceptibility to multiple autoimmune and infectious diseases (2, 3, 4). PTPN22 has been implicated in the inhibition of T cell receptor (TCR) signaling in effector T cells *via* dephosphorylation of positive regulatory tyrosines in the activation domain of PTKs such as lymphocyte-specific protein tyrosine kinase (LCK) and zeta-chain–associated protein kinase 70 (ZAP70) in a phosphatase activity–dependent manner (5, 6, 7). Moreover, PTPN22 promotes type I interferon release in myeloid cells by regulating lysine 63 (K63)-linked ubiquitination (8) of TNF receptor-associated factor 3 (TRAF3). Many types of protein kinases and phosphatases act in concert with PTPN22 to orchestrate TCR signaling following stimulation (2). Among these, protein kinase C (PKC) acts as a cytoplasmic signal mediator in TCR signaling in part by controlling the function of PTPN22. Specifically, phosphorylation on PTPN22 Ser^751^ inhibits lysine 48 (K48)-linked ubiquitination to prolong its half-life, while impairing its recruitment to the plasma membrane and enhancing its interaction with C-terminal Src kinase (9). Therefore, phosphorylation of PTPN22 has been shown to act as a molecular rheostat that modulates its inhibitory effect on TCR signaling (9, 10, 11). Notably, a C1858T SNP, corresponding to an arginine (R) to tryptophan (W) mutation at position 620 in the P1 proline-rich motif of PTPN22 leads to reduced binding to C-terminal Src Kinase and increased risk of several autoimmune diseases (12, 13).

As a known negative regulator in T cells, the enzymatic activity of PTPN22 is essential for mediating T cell immune responses (2, 7, 14). The classic PTP catalytic domain (amino acids 1–300), located at the N-terminal end of the protein, has been structurally and functionally characterized in detail. It is followed by a regulatory interdomain (amino acids 301–600) and a C-terminal domain containing four putative proline-rich motifs (P1–P4), which govern its phosphatase activity in cells by mediating protein–protein interactions (15, 16). However, the interdomain has not been thoroughly characterized, and its mechanism of action remains poorly understood at both the functional and molecular levels, despite the growing interest in the therapeutic exploitation of the allosteric regulation of PTPs. Here, we report the identification of a novel phosphorylation site at Ser^325^ in the PTP domain-proximal region of the interdomain of PTPN22 by phospho-mass spectrometry (MS) which is not conserved in rodents. By analyzing the effects of Ser^325^ mutations in CRISPR/Cas9 engineered cells and in purified truncated proteins, we propose that Ser^325^ phosphorylation regulates the phosphatase activity of PTPN22 in cells, thus altering its effect on downstream signaling. Our findings reveal a previously unappreciated mechanism for regulating the abundance of active PTPN22 in T cells.

## Results

### PTPN22 Ser^325^ is an inducible phosphorylation site in human T cells downstream of GSK3

**Figure 1:**
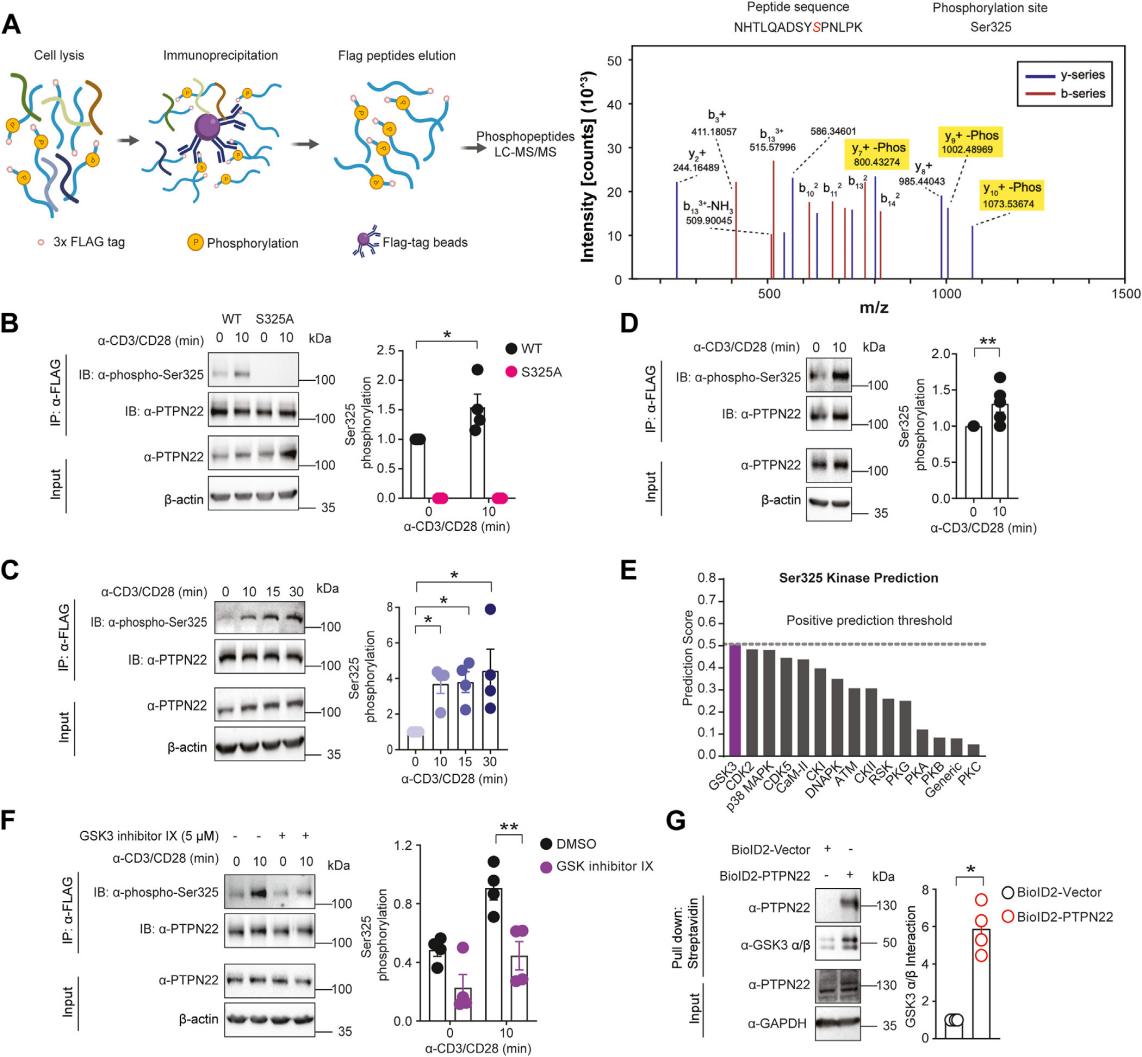
**PTPN22 Ser**^**325**^**is an inducible GSK3 phosphorylation site in human T cells.***A*, schematic illustration of 3× FLAG PTPN22 protein purification (*left panel*) and mass spectra (*right panel*) indicating Ser^325^ phosphorylation in immunoprecipitated PTPN22 from 3× FLAG PTPN22 Jurkat WT cells cross-linked with antibodies against human CD3/CD28. Data is representative of three independent biological replicates. Peptide MS2 fragmentation pattern shown displaying *m/z* and peptide spectral match intensity. *B*, Western blot analysis obtained using a phospho-Ser^325^–specific antibody in immunoprecipitated PTPN22 from the lysates of PTPN22 KO Jurkat cells overexpressing 3× FLAG WT or S325A PTPN22 and treated with antibodies against human CD3/CD28 for the indicated time (*left panel*). Quantification of the phospho-Ser^325^/total PTPN22 ratio normalized to nonstimulated conditions in four independent experiments (*right panel*). Statistical significance was assessed using the two-way ANOVA test followed by Bonferroni’s post hoc test, ∗ *p* < 0.05. *C*, endogenous Ser^325^ phosphorylation was analyzed by Western blotting in PTPN22 immunoprecipitated from lysates of 3× FLAG WT PTPN22 Jurkat cells stimulated with antibodies against human CD3/CD28 for the indicated times (*left panel*). Quantifications of the phospho-Ser^325^/total PTPN22 ratio normalized to nonstimulated conditions in four independent experiments (*right panel*). Statistical significance was assessed using the Kruskal–Wallis test, ∗*p* < 0.05. *D*, immunoprecipitation analysis of endogenous PTPN22 Ser^325^ phosphorylation in lysates of human primary CD4^+^ effector T cells stimulated with antibodies against human CD3/CD28 for the indicated time (*left panel*). Quantification of the phospho-Ser^325^/total PTPN22 ratio normalized to nonstimulated conditions in five independent experiments (*right panel*). Statistical significance was assessed by using the Kolmogorov–Smirnov test, ∗∗ *p* < 0.01. *E*, prediction of potential kinases responsible for PTPN22 Ser^325^ phosphorylation in descending order from *left to right*. *F*, immunoprecipitation analysis of phospho-PTPN22 Ser^325^ in lysates of 3× FLAG PTPN22 WT Jurkat cells with or without incubation with 5 μM GSK3 inhibitor IX and stimulation with antibodies against human CD3/CD28 (*left panel*). Quantifications of phospho-Ser^325^/total PTPN22 ratio from Western blot analysis of four independent experiments (*right panel*). Statistical significance was assessed using the two-way ANOVA test followed by Bonferroni’s post hoc test, ∗∗*p* < 0.01. *G*, immunoprecipitation analysis of PTPN22 and GSK3 interaction in lysates of BioID2 Jurkat cells (*left panel*). Histograms shows quantifications of immunoprecipitated GSK3 α/β based on the Western blot analysis and are representative of four independent experiments (*right panel*). Statistical significance was assessed using the Kolmogorov–Smirnov test, ∗*p* < 0.05. CD, cluster of differentiation; GSK3, glycogen synthase kinase 3; PTPN22, protein tyrosine phosphatase nonreceptor type 22.

Previously, we reported on the identification by unbiased phospho-MS–based analysis of a novel phosphorylation site at Ser^751^ in the C-terminal domain of PTPN22 purified from HEK293T cells treated with the serine/threonine phosphatase inhibitor calyculin A and its function in the regulation of PTPN22 (9). To assess potential roles of PTPN22 phosphorylation in T cells following TCR stimulation, 3× FLAG-tagged PTPN22 protein was purified from Jurkat cells treated with antibodies against human cluster of differentiation (CD) 3 and 28 and cross-linked with a goat anti-mouse immunoglobulin (Ig) and then subjected to phospho-MS analysis. Lysates from PTPN22 KO cells (9) were used as a negative control (Fig. S1*A*).). As shown in Figure 1*A*, a novel phosphorylation site at Ser^325^ in the interdomain of PTPN22 was observed. To validate the site, a serine to alanine mutant was overexpressed in Jurkat PTPN22 KO cells, with WT PTPN22 serving as a control. As expected, Ser^325^ phosphorylation in PTPN22 immunoprecipitated from cells overexpressing PTPN22 S325A was fully abrogated in cells treated with or without anti-CD3/CD28 antibodies, as assessed by Western blots using a phospho-Ser^325^–specific antibody. In contrast, Ser^325^ phosphorylation was detected in immunoprecipitated WT PTPN22 and enhanced after TCR engagement (Fig. 1*B*). Sequence alignment around Ser^325^ indicates this site is well conserved in PTPN22 across various species with the notable exception of rodents (Fig. S1*B*). Next, sustained Ser^325^ phosphorylation of endogenous PTPN22 was confirmed in 3× FLAG PTPN22 KI Jurkat cells (9), showing increasing phosphorylation in a time course of TCR stimulation over 30 min (Fig. 1*C*). Moreover, Ser^325^ phosphorylation also occurred in human primary CD4^+^ effector T cells after 10 min of TCR stimulation (Fig. 1*D*).

We next focused our attention on the potential protein kinases responsible for the phosphorylation of PTPN22 at Ser^325^. First, *in silico* prediction methods were used to query kinases likely to catalyze this posttranslational modification (17, 18). As shown in Figure 1*E*, glycogen synthase kinase 3 (GSK3) was identified as the top candidate, followed by cyclin-dependent kinase 2 and p38 mitogen-activated protein kinase. To validate these results, 3× FLAG WT PTPN22 Jurkat cells were treated with or without a specific GSK3 inhibitor and analyzed for TCR-induced phosphorylation of Ser^325^. Consistent with the prediction results, upregulation of PTPN22 Ser^325^ phosphorylation upon TCR stimulation was substantially diminished in cells treated with 5 μM GSK3 inhibitor IX (Fig. 1*F*). Dose-dependent inhibition shows 5 μM inhibitor concentration was sufficient to suppress Ser^325^ phosphorylation after TCR stimulation (Fig. S1*C*). In contrast, no statistically significant changes were observed in cells treated with increasing concentrations of p38 mitogen-activated protein kinase inhibitor (Fig. S1*D*). Next, we probed the interaction between PTPN22 and GSK3 in Jurkat T cells. To bypass the problem of nonspecific protein binding in immunoprecipitation (IP) assays, PTPN22 KO Jurkat cells stably expressing BioID2 empty vector or BioID2-PTPN22 WT were constructed and used to analyze PTPN22-protein interactions by proximity labeling (19). Biotinylated proteins were pulled down on Streptavidin beads and detected by Western blotting with horseradish peroxidase–conjugated Streptavidin (Fig. S1*E*). Significantly more biotinylated GSK3 α/β was pulled down in cells transfected with PTPN22 than with empty vector (Fig. 1*G*), suggesting the presence of substantial interaction between these two proteins. Taken together, these results show that Ser^325^ is a phosphorylation site of PTPN22 in human T cells downstream of GSK3 and is inducible upon TCR stimulation.

### Ser^325^ phosphorylation is involved in the regulation of TCR signaling

**Figure 2:**
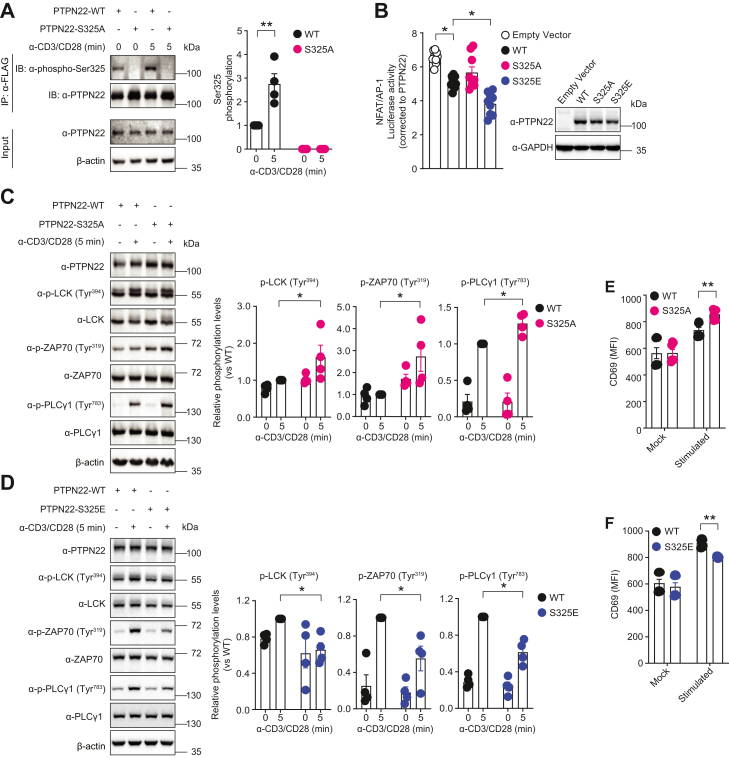
**Phosphorylation of PTPN22 Ser**^**325**^**enhances the inhibitory effect of PTPN22 on T cell receptor signaling.***A*, immunoprecipitation analysis of PTPN22 Ser^325^ phosphorylation in lysates of 3× FLAG PTPN22 WT and CRISPR/Cas9-mediated S325A KI Jurkat cells stimulated with antibodies against human CD3/CD28 for the indicated time by Western blotting (*left panel*). Histogram shows quantifications of the phospho-Ser^325^/total PTPN22 ratio normalized to nonstimulated condition and is representative of four independent experiments (*right panel*). Statistical significance was assessed using the two-way ANOVA followed by Bonferroni’s post hoc test, ∗∗*p* < 0.01. *B*, dual-luciferase reporter assay analysis of full-length PTPN22 inhibition of TCR signaling in PTPN22 KO Jurkat cells overexpressing full-length 3× FLAG WT, S325E, or S325A PTPN22 together with NFAT/AP-1 firefly and *Renilla* luciferase reporters and stimulated with antibodies against human CD3/CD28. Luciferase activity was measured (*left panel*), and the numbers on the *y*-axis indicate NFAT/AP-1 firefly luciferase activity normalized first to *Renilla* luciferase activity in each group (KO, WT, or S325 mutant), then to the amount of PTPN22 relative to that of GAPDH as assessed by Western blotting (*right panel*). Mean ± SEM are shown from three independent experiments each with three replicates per condition. Statistical significance was assessed by using the Kruskal–Wallis test, ∗*p* < 0.05. *C* and *D*, Western blot analysis of TCR signaling in 3× FLAG PTPN22 WT, and CRISPR/Cas9 mediated S325A (*C*) or S325E (*D*) KI Jurkat cells treated with antibodies against human CD3/CD28 for indicated time, followed by detection of phosphorylated LCK (Tyr^394^), ZAP70 (Tyr^3^^19^), and PLC-γ (Tyr^783^) in lysates (*left panels*). Histograms show quantification of phosphorylated LCK, ZAP70, and PLC-γ normalized to relative total protein by four independent experiments (*right panels*). Statistical significance was assessed using the Kolmogorov–Smirnov test, ∗*p* < 0.05. *E* and *F*, flow cytometry analysis of TCR-induced CD69 expression in 3× FLAG PTPN22 WT, S325A (*E*), or S325E (*F*) KI Jurkat cells treated with (stimulated) or without (mock) antibodies against human CD3/CD28 for 4 h. Histograms show median fluorescent intensity (MFI) from seven independent experiments. Statistical significance was assessed using two-way ANOVA, followed by Bonferroni’s post hoc test, ∗∗*p* < 0.01. AP-1, activator protein-1; CD, cluster of differentiation; GSK3, glycogen synthase kinase 3; LCK, lymphocyte-specific protein tyrosine kinase; NFAT, nuclear factor of activated T cells; PLC, phospholipase C; PTPN22, protein tyrosine phosphatase nonreceptor type 22; TCR, T cell receptor; ZAP70, zeta-chain–associated protein kinase 70.

Having shown that PTPN22 Ser^325^ phosphorylation levels were increased following TCR stimulation, we hypothesized that phosphorylation on this residue regulates the inhibitory effect of PTPN22 in TCR signaling. To assess this, we introduced mutations of Ser^325^ in the endogenous protein *via* CRISPR/Cas9-mediated homozygous mutagenesis. Based on the chemical properties of amino acid side chains, the serine (S) to alanine (A) mutation will abolish phosphorylation, while glutamic acid (E) is predicted to mimic the phosphorylated state of serine (20, 21). Therefore, CRISPR/Cas9 editing was applied on 3× FLAG PTPN22 WT Jurkat cells to obtain homozygous 3× FLAG-tagged PTPN22 KI Jurkat cell lines carrying the S325A or S325E mutation (Fig. S2, *A* and *B*). The mutants were confirmed by complementary DNA sequencing (Fig. S2*C*). Western blotting against PTPN22 in cell lysates of different KI lines confirmed they express PTPN22 at a similar level as the original PTPN22 WT cells (Fig. S2*D*). As expected, endogenous PTPN22 immunoprecipitated from PTPN22 WT cells exhibited increasing Ser^325^ phosphorylation upon CD3/CD28 costimulation, while phosphorylation in PTPN22 S325A cells was undetectable by the phospho-Ser^325^–specific antibody even under stimulation by CD3/CD28 antibodies (Fig. 2*A*), confirming the pattern previously observed in PTPN22 KO Jurkat cells overexpressing WT and S325A PTPN22 (Fig. 1*B*).

PTPN22 is known to act as an inhibitor of TCR signaling in effector T cells by dephosphorylating key tyrosine kinases (2). To understand the effect of Ser^325^ phosphorylation in the context of TCR signaling, we first measured the nuclear factor of activated T cells and activator protein-1 (NFAT/AP-1) luciferase reporter activities after TCR stimulation in PTPN22 KO Jurkat cells overexpressing various PTPN22 full-length constructs. Cells overexpressing PTPN22 S325E mutant showed a gain-of-function phenotype of PTPN22, as measured by significantly reduced luciferase reporter activities compared to cells overexpressing PTPN22 WT, while cells overexpressing PTPN22 S325A mutant exhibited a modest but significant increase in activation of luciferase reporters, indicating a loss-of-function effect on PTPN22 (Fig. 2*B*). Next, we assessed the phosphorylation levels of several upstream mediators of signaling pathway in PTPN22 WT or mutants KI Jurkat cell lines. Compared to WT, phosphorylation of LCK Tyr^394^, ζ chain–associated protein tyrosine kinase of 70 kDa (ZAP70) Tyr^3^^19^, and phospholipase C γ (PLC-γ) Tyr^783^ were significantly increased in S325A KI cells upon 5-min TCR stimulation, consistent with a loss-of-function of PTPN22 (Fig. 2*C*). In contrast, phosphorylation of the same kinases was reduced in S325E KI cells, as expected for a gain-of-function phenotype in regulating TCR signaling (Fig. 2*D*). A similar trend was observed in PTPN22 KO Jurkat cells overexpressing PTPN22 WT, S325A (Fig. S2*E*), or S325E (Fig. S2*F*) costimulated by human anti-CD3/CD28 antibodies. Moreover, activation of T cells was also assessed in WT and PTPN22 mutant KI cells by quantifying expression of the T cell activation marker CD69 (22). According to flow cytometry analysis, expression of CD69 was substantially increased in PTPN22 S325A KI Jurkat cells (Fig. 2*E*) and inhibited in S325E KI cells when compared to WT cells (Fig. 2*F*) after TCR stimulation. To exclude potential confounding effects of CD3 expression that might alternatively explain the observed differences in CD69 levels, the expression of CD3 was analyzed and found to be similar between WT, S325A, and S325E KI cells under TCR stimulation (Fig. S2, *G* and *H*). Thus, Ser^325^ phosphorylation is an important modification on PTPN22 that contributes to the inhibitory regulation of TCR-dependent signal transduction by PTPN22.

### Ser^325^ phosphorylation promotes alternative signaling pathways

**Figure 3:**
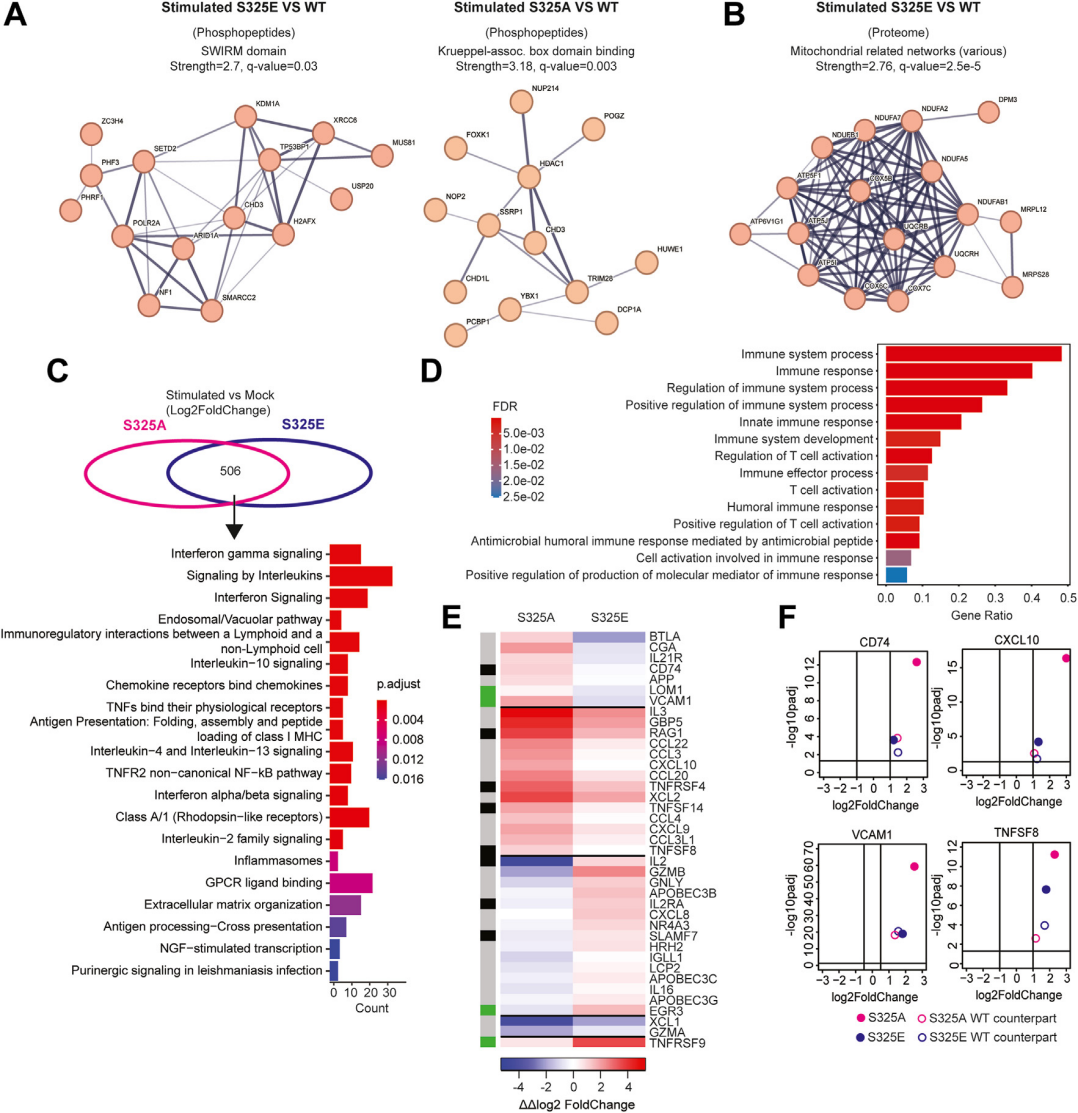
**Signaling pathway changes in global phospho-MS and bulk RNA-seq.***A*, STRING-DB–generated protein–protein interaction networks of proteins with enriched phosphopeptides from stimulated S325E/A conditions further controlled by removing features with similar increases under mock stimulation. Only terms >2 in strength and an adjusted *p* value <0.05 were considered. *B*, STRING-DB–generated protein–protein interaction networks of proteins increased in stimulated S325E. Of note, S325A exhibited no enrichments. *C*, overrepresentation analysis for reactome pathways. Five hundred six DEGs (≥2-fold and adjusted *p* value < 0.05: stimulated *versus* mock) in both S325A and S325E. *D*, GO functional enrichment for biological process of top genes from 506 DEGs in *C* ranked by ΔΔlog2FoldChange (99 genes: ΔΔlog2FoldChange>1). Selected GO terms containing “immune” and “T cell activation” terms displayed. *E*, Δlog2(FoldChange) values for genes from GO terms “immune response” (*gray*), “T cell activation” (*green*), or both (*black*). *F*, *volcano plots* (stimulated *versus* mock) for S325A, S325E, and their WT counterparts. Exemplar genes *CD74* and *CXCL10* are shown. DEG, differentially expressed gene; GO, gene ontology; MS, mass spectrometry.

To further explore the consequences of Ser^325^ phosphorylation at a systems level, we compared stimulated S325E and S325A KI cells to their WT counterpart using MS-based phosphoproteomics at 5 min of T cell activation by TCR engagement (Fig. S3*A*). While controlling for mock stimulation background, phosphopeptides enriched in both S325E and S325A cells were associated with alterations in histone status/chromatin modification. Phosphopeptides from S325E cells were significantly enriched in proteins associated with Swi3p, Rsc8p, and Moira domain binding, while S325A’s strongest association was to the Krueppel-associated box domain binding (Fig. 3*A*). The Swi3p, Rsc8p, and Moira domain is largely associated with increased transcriptional activity, while Krueppel-associated box domain is broadly associated with its repression (23, 24). This suggests that, as constitutively phosphorylated and unphosphorylated versions of Ser^325^ alter downstream transcriptional activity, they potentially regulate alternate pathways and affect the cell state differentially. Downstream effects of this alternative phospho-status were also present when comparing the underlying shotgun proteomes, where S325E revealed a strong enrichment in proteins associated with mitochondrial activity, while S325A had no obvious enrichments (Fig. 3*B*).

Next, to investigate how Ser^325^ phosphorylation affects downstream transcriptional activity during T cell activation, we carried out bulk mRNA transcriptomics under CD3/CD28 antibodies or mock stimulation (Fig. S3*A*). The results showed a total of 912 genes and 665 genes were differentially expressed in S325A (unphosphorylated PTPN22) or S325E (constitutively phosphorylated PTPN22) cells under TCR stimulation, respectively (FoldChange >2 and adjusted *p* value <0.05) when comparing the transcriptomes of stimulated cells with mock. Further enrichment analysis showed differences in the enriched pathways associated with those differentially expressed genes (DEGs) unique to each group (Fig. S3*B*). 506 DEGs were shared by these two groups (Fig. 3*C*), and these genes were mainly enriched in cytokines (interleukins, interferon), chemokines, antigen presentation, and G-protein-coupled receptor ligand binding pathways, where PTPN22 is known to act as a key regulator (2, 25, 26, 27). Moreover, from these 506 DEGs, those having divergent responses were obtained as defined by ΔΔlog2(FoldChange) >1 and adjusted *p* value <0.05 between the S325A and S325E groups (top 99 genes from 506 genes in Fig. 3*C*). Functional enrichment analysis based on gene ontology biological process showed 99 genes were enriched in pathways related to immune system processes, immune responses, and T cell activation (Fig. 3*D*). Furthermore, all genes related to the gene ontology terms *Immune response* and *T cell activation* were analyzed by controlling for the WT stimulation background and showed divergent regulation between S325A and S325E groups indicating a loss- or gain-of function of PTPN22 in T cells (Fig. 3*E*). Surprisingly, the CD74 gene, which regulates T cell development and plays important roles in many inflammatory diseases (28), was reduced in the S325E group relative to the S325A group. The transcription of early growth response 3 (EGR3), a key negative regulator of T cell activation (29), was increased in the S325E group relative to the S325A group (Fig. 3, *E* and *F*). Interestingly, multiple chemokine genes, including CXCL10, were highly upregulated in the S325A group. Tumor necrosis factor receptor superfamily member 9 gene (also known as CD137), which is expressed on activated T cells and is a promising new target for immunotherapy (30), was highly induced in the S325E group (Fig. 3, *E* and *F*). These results resemble the phenotype of the PTPN22 1858TT (R620W) allele, which reduces the production of CXCL10 in peripheral blood mononuclear cells stimulated with poly (I:C) (31) and promotes tumor necrosis factor receptor superfamily member 9 expression in CD4^+^ T cells after TCR triggering (32). In summary, Ser^325^ phosphorylation modifies T cell activity by altering the expression of chemokines and T cell regulators.

### *In vitro* effects of mimicking Ser^325^ phosphorylation on the catalytic activity of PTPN22

**Figure 4:**
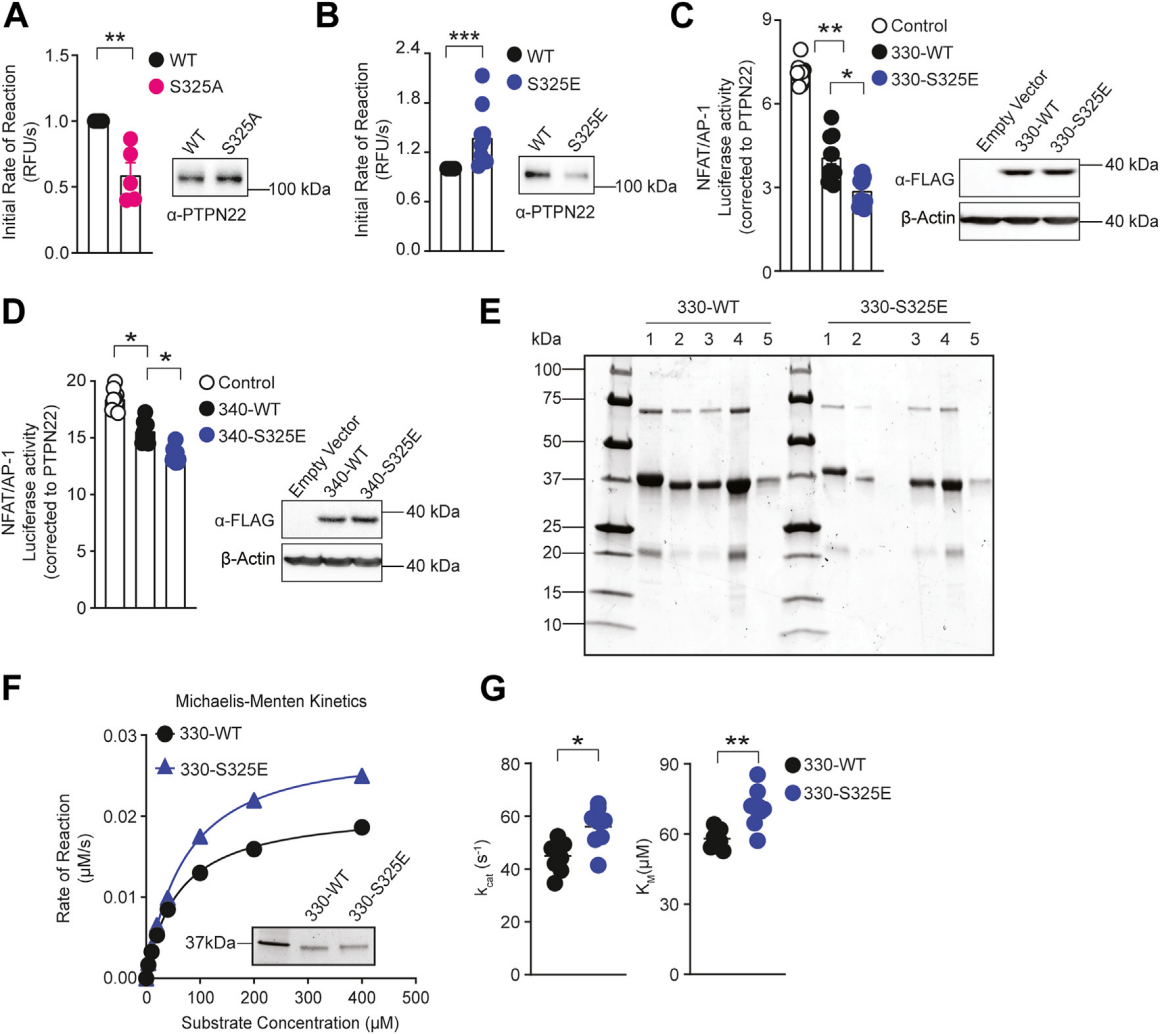
***In vitro* catalytic activity of PTPN22 and its variants.***A* and *B*, phosphatase activity assays were performed using immunoprecipitated PTPN22 from PTPN22 WT, or S325A, S325E KI Jurkat cells, and DiFMUP as a substrate. Histograms (*left panel*) show quantification of initial rates of reaction normalized to the amount of PTPN22 WT as assessed by Western blotting (*right panel*). Mean ± SEM are shown from five (*A*) and ten (*B*) independent experiments. Statistical significance was assessed by using the Kolmogorov–Smirnov test, ∗∗ *p* < 0.01, ∗∗∗ *p* < 0.001. *C* and *D*, dual-luciferase reporter assay analysis of truncated WT and S325E inhibition of TCR signaling in PTPN22 KO Jurkat cells overexpressing 3 × FLAG PTPN22_1-340_ (*C*), or PTPN22_1-330_ (*D*) WT, and S325E together with NFAT/AP-1 firefly and *Renilla* luciferase reporters and stimulated with antibodies against human CD3/CD28. Luciferase activity was measured and quantified as described in Figure 2*B*. Means ± SEM are shown from three or four independent experiments each with three replicates per condition. Statistical significance was assessed by using the Kruskal–Wallis test, ∗*p* < 0.05, ∗∗ *p* < 0.01. *E*, representative SDS-PAGE of final purified samples of PTPN22_1-330_ WT and S325E recombinant proteins. *F* and *G*, phosphatase activity assays were performed by using recombinant PTPN22_1-330_ WT or S325E and DiFMUP as a substrate. *F*, representative Michaelis–Menten curve of eight independent experiments each with three technical replicates and representative SDS PAGE of 1 μM PTPN22_1-330_ WT and S325E recombinant proteins. *G*, *dot plot* showing *k*_*cat*_*and K*_*M*_. Each data point represents one of eight independent experiments performed as in (F). Statistical significance was assessed using two-tailed Mann–Whitney test, ∗ *p* < 0.05, ∗∗ *p* < 0.01. AP-1, activator protein-1; CD, cluster of differentiation; DiFMUP, 6,8-difluoro-4-methylumbelliferyl phosphate; NFAT, nuclear factor of activated T cells; PTPN22, protein tyrosine phosphatase nonreceptor type 22; TCR, T cell receptor.

Having shown that Ser^325^ phosphorylation promotes PTPN22 cellular activity to inhibit TCR signaling, we postulated that it directly affects the enzyme’s catalytic activity. To confirm this, a series of *in vitro* assays were performed. First, phosphatase activity differences between full-length WT and mutant PTPN22 were studied using immunoprecipitated PTPN22 and 6,8-difluoro-4-methylumbelliferyl phosphate (DiFMUP) as a substrate. Protein immunoprecipitated from PTPN22 S325A KI Jurkat cells showed a 2-fold reduced initial rate of reaction compared to the WT protein (Fig. 4*A*), suggesting decreased phosphatase activity of the S325A mutant. Conversely, the S325E mutant showed a small (approximately 1.2-fold) increased enzymatic activity (Fig. 4*B*), possibly due to already high Ser^325^ phosphorylation levels in the WT protein. Ser^325^ is located in the interdomain of PTPN22 proximal to the catalytic domain (1–300). In order to better understand the role of Ser^325^ phosphorylation, the behavior of WT and mutant PTPN22 was studied *in vitro* using bacterially purified proteins. As sufficient amounts of highly purified full-length PTPN22 could not be obtained, we decided to use truncated PTPN22 to investigate whether mimicking Ser^325^ phosphorylation modulates its phosphatase activity by directly affecting the catalytic domain. In order to do this, we first confirmed that the S325E mutation had similar effects on truncated PTPN22’s regulation of TCR signaling as on full-length PTPN22. Thus, TCR-induced NFAT/AP-1 activation was examined in PTPN22 KO Jurkat cells overexpressing WT and S325E mutant PTPN22_1-330_ and PTPN22_1-340_. As shown in Figure 4, *C* and *D*, the truncated PTPN22 variants recapitulate the trend observed above for the full-length proteins in the luciferase reporter assay, with truncated PTPN22 S325E displaying gain-of-function inhibition of TCR signaling. Next, the catalytic activities of bacterially expressed and purified recombinant PTPN22_1-330_ or PTPN22_1-340_ variants were measured (Figs. 4*E* and S4*A*). When using purified recombinant PTPN22_1-330_ in kinetic assays with DiFMUP, we observed higher k_cat_ and K_*M*_ values for S325E than WT PTPN22 (Fig. 4, *F* and *G*). Recombinant PTPN22_1-340_ S325E exhibited a somewhat statistically elevated *k*_*cat*_ compared to the WT when using DiFMUP as substrate, while the effect on K_*M*_ was not significant (Fig. S4, *B* and *C*). The ∼1.2-fold difference in k_cat_, however, was much smaller than would be expected on the basis of the kinetic data using immunoprecipitated protein and the effect on cellular signaling. Taken together, these results suggest Ser^325^ phosphorylation enhances PTPN22 enzymatic activity in T cells; however, the effect appears to be dependent on the cellular environment *via* an as yet unknown mechanism.

### Mimicking Ser^325^ phosphorylation affects amino acids around the active site

**Figure 5:**
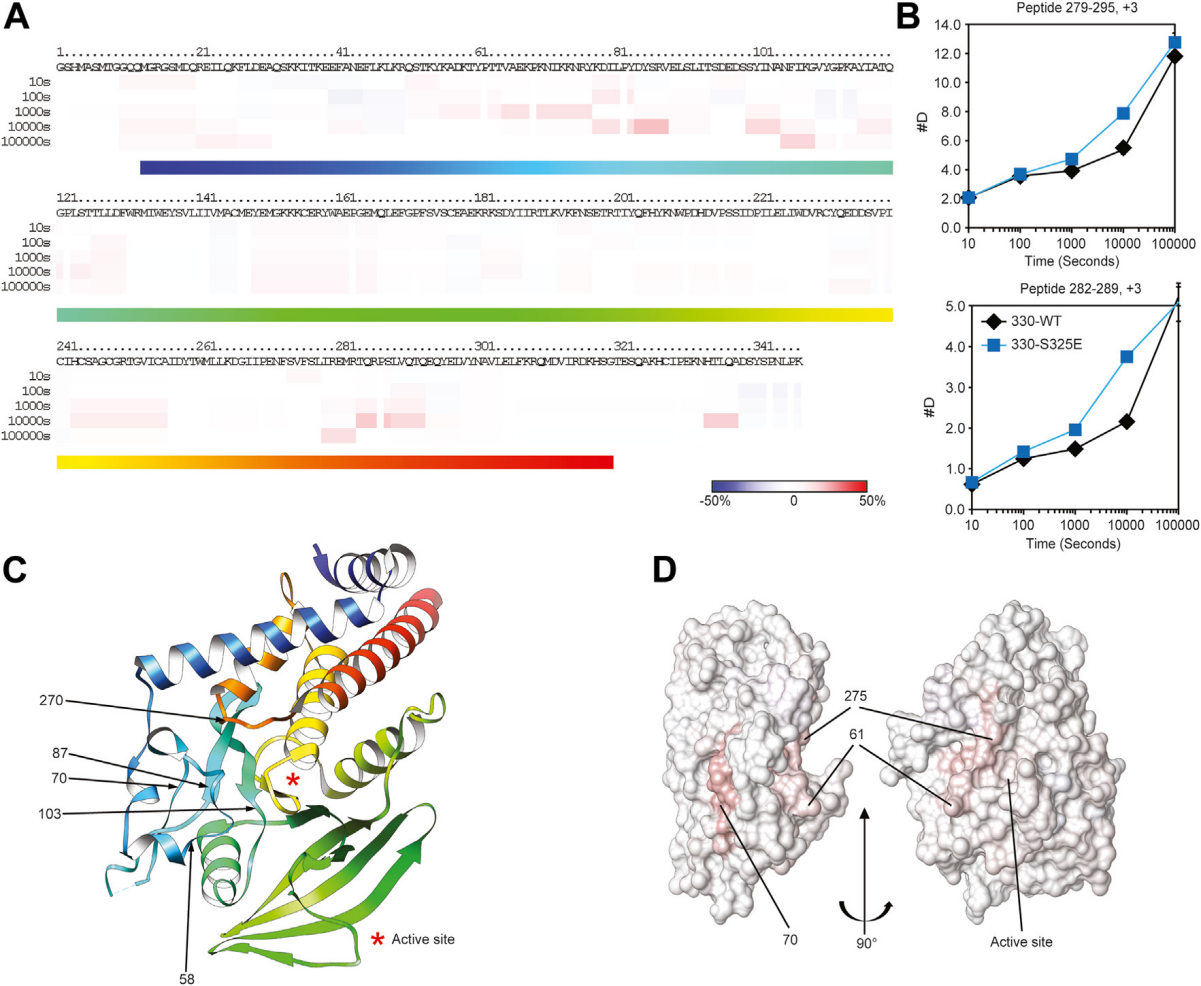
**Effect of Ser**^**325**^**phosphorylation mimic on the deuterium exchange rates of PTPN22 1 to 330.***A*, *rainbow plot* showing percent differences in deuterium exchange between PTPN22_1-330_ WT and S325E. Deuterium exchange was assessed for five different time points from 10 to 100000 s. The numbering of the polypeptide chain follows the sequence of the protein as used in the experiment, which has a 17-amino acid N-terminal leader preceding the native methionine 1. Data are representative of two independent experiments. *B*, the averages of number of deuterium exchanged (#D) shown over time for two of the peptides with the greatest changes. *C*, *ribbon representation* of PTPN22 (PDB code 2P6X) annotated with the location of the active site and of polypeptide regions mentioned in the text. For reference, the ribbon color matches the bottom band in the sequence view in A. *D*, solvent accessible surface representation of the PTP domain in two orthogonal views colored according to the percent difference in the exchange rate at 10,000 s as shown in A. Key residues are indicated. The molecular graphics objects in (*C*) and (*D*) were generated with UCSF Chimera (57). Ni-NTA, nickel-nitrilotriacetic acid; PTP, protein tyrosine phosphatase; PTPN22, PTP nonreceptor type 22.

As Ser^325^ phosphorylation may contribute to the regulation of PTPN22 catalytic activity, we set out to probe whether any regions of the PTP domain were specifically affected by the Ser^325^ substitution using recombinant proteins (Fig. 4*E*) *in vitro*. Deuterium exchange (DX)-MS detects, and allows to map, changes in conformation, solvent accessibility, or dynamics in a protein due for example to ligand binding, chemical modification, or mutation (33). In this method, DX of amide protons under controlled conditions for specified time intervals is followed by nonspecific proteolytic digestion and mass spectrometric analysis of the resulting peptide collection. As shown in Figure 5, we compared the exchange rates for peptides spanning the entire sequence for PTPN22_1-330_ WT and S325E. The rainbow plot in Figure 5*A*, representative of the findings from two independent repeats, shows mapping of faster and slower exchanging regions along the sequence upon introduction of the phosphorylation mimic. Interestingly, multiple regions exchanged faster in the S325E mutant. Close direct analysis of DX plots, two examples of which are shown in Figure 5*B*, along the entire sequence in both repeats revealed that peptides containing five segments, approximately centered around residues 58, 70, 87, 103, 270, consistently show measurable increases in the observed DX rates in the intermediate/slow range (Fig. 5*C*). While the differences were small, when mapped on the three-dimensional structure of the PTP domain of PTPN22, these regions form a cluster connecting the protein surface to the active site, though the residues most directly involved in catalysis appear to be unaffected (Figs. 5*D* and S4, *D* and *E*). Notably, the C-terminal amino acids exhibit fast exchange rates and are little affected by the mutation, suggesting that the interaction with their PTP domain might be dynamic in nature.

### Amino acid residues proximal to the PTP domain are most affected by Ser^325^ phosphorylation

**Figure 6:**
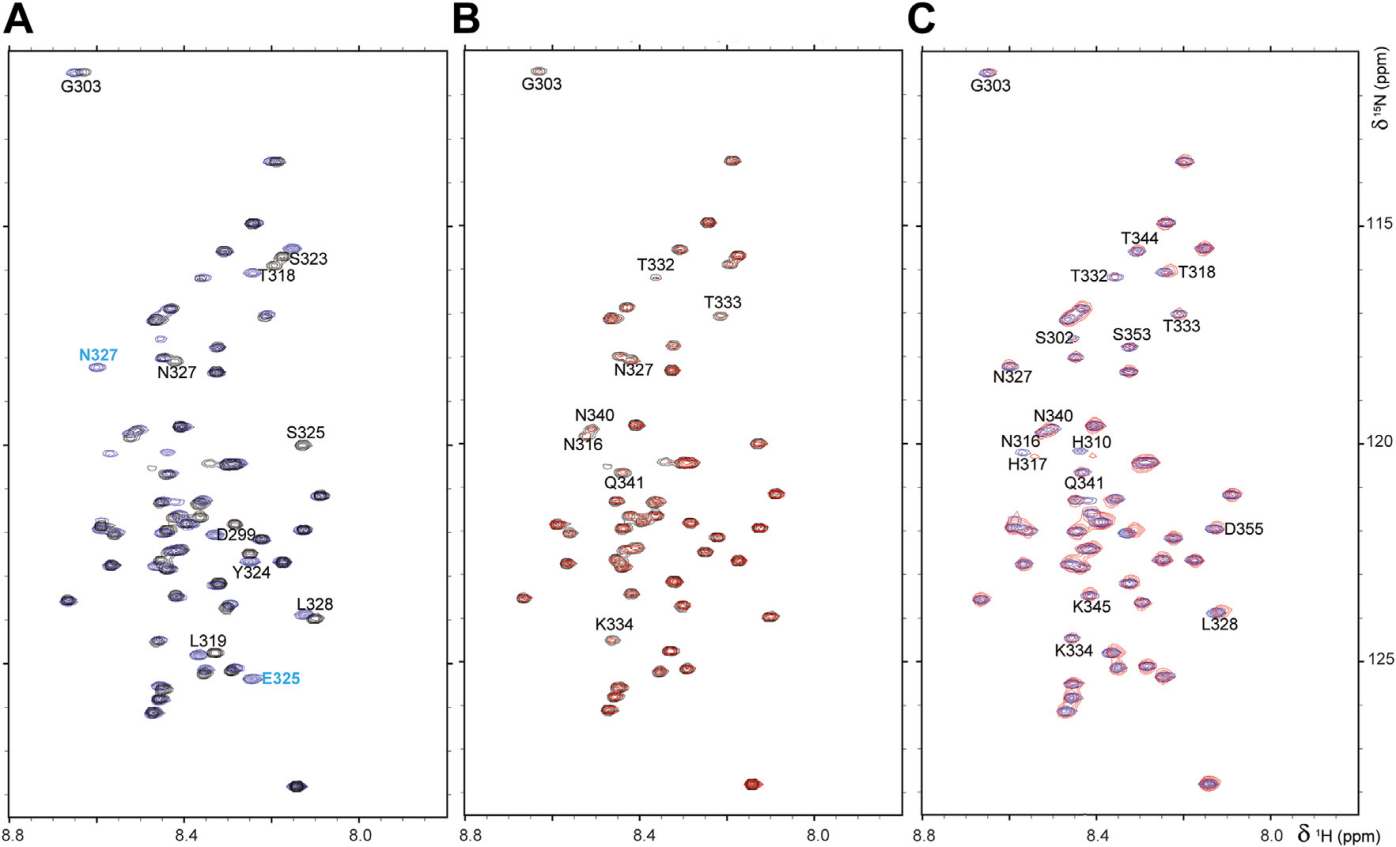
**Effect of mimicking Ser**^**325**^**phosphorylation on the interaction between the PTPN22 interdomain and catalytic domain.***A*, 2D [^1^H, ^15^N] HSQC spectrum of ^15^N-labeled PTPN22 interdomain (residues 299–360, *black*) and S325E (*blue*). S325 and E325 residues are annotated. *B*, 2D [^1^H, ^15^N] HSQC spectrum of ^15^N-labeled PTPN22 interdomain (residues 299–360) alone (*black*) and in complex with the PTPN22 catalytic domain (residues 1–299; *red*). Peaks with changing intensities are annotated. *C*, 2D [^1^H, ^15^N] HSQC spectrum of ^15^N-labeled PTPN22 S325E interdomain (residues 299–360) alone (*blue*) and in complex with the PTPN22 catalytic domain (residues 1–299; *red*). Peaks with changing intensities are annotated. HSQC, heteronuclear single quantum coherence; PTPN22, protein tyrosine phosphatase nonreceptor type 22.

Finally, we used NMR spectroscopy to investigate the PTPN22 interdomain interaction with the catalytic domain. Unfortunately, the yields of purified PTPN22 are low (2–3 mg per liter of *Escherichia coli* cell culture) and the necessary isotope labeling (especially deuteration) let to a yield of 0.5 to 1 mg per liter (despite careful bacterial adaptation), making it impossible to perform detailed NMR spectroscopy on the PTPN22 catalytic domain. Thus, we used an alternative approach, where we expressed and purified the PTPN22 catalytic domain (residues 1–299) and interdomain residues 299 to 360 separately. This approach has previously been successfully applied to the study of PTPN2 (34). The PTPN22 interdomain can be produced in high yields and labeled for NMR spectroscopy studies. We performed a sequence specific backbone assignment of PTPN22 299 to 360 and directly compared 2D [^1^H,^15^N] heteronuclear single quantum coherence (HSQC) spectrum of the WT PTPN22 interdomain with that of the S325E variant. Both spectra showed all hallmarks of an intrinsically disordered region (IDR), including limited chemical shift dispersion in the ^1^H dimension, due to a lack of hydrogen bonds in secondary structure elements (Fig. 6*A*). As expected, residues adjacent to the 325 position showed chemical shift changes and, interestingly, N-terminal interdomain residues were also unexpectedly affected by the charge on S325E, indicating a change in the global ensemble of structures. It is unclear if these effects are limited to the PTPN22 interdomain peptide or also present in the protein. We then repeated these data in the presence of a large excess of PTPN22 catalytic domain (Fig. 6*B*). The peak intensities of a subset of interdomain residues decreased in the presence of the catalytic domain, indicative of a weak interaction between the PTPN22 interdomain and the catalytic domain. We saw a similar change in peak intensities with WT and the S325E variant (Fig. 6*C*). This demonstrates that both the WT and the S325E variant of the PTPN22 interdomain interact with the PTPN22 catalytic domain.

## Discussion

Nonreceptor PTPs generally comprise a classic PTP domain and one or more regulatory domains. In contrast to the high conservation of the former, the diversity in the sequences, structures, and modes of action of the latter has led to the view that they could be successfully targeted for drug development. Attempts at elucidating the mechanisms by which the interdomain of PTPN22, and other NT4 subclass PTPs, control the protein’s activity have however been set back by both its predicted lack of an ordered structure and the difficulty in obtaining adequate amounts of purified protein. In turn, this has limited progress in the study of the regulation of PTPN22 and its consequences in cells. In this context, the presence of sites of posttranslational modification in the interdomain presents additional challenges but also a potential approach to investigate the function and mechanism of allosteric regulation of PTPN22. We had previously shown that the PTP-proximal region of the interdomain negatively affects the catalytic activity of PTPN22 *in vitro via* an intramolecular mechanism (16). In this report, we add another piece to the puzzle by showing that phosphorylation at Serine 325, which is induced in T cells upon TCR stimulation, can further fine-tune this effect. The work presented in this paper points to a few possible ways to approach the selective drugging of PTPN22.

We identified a region of the interdomain, proximal to the PTP domain, which can modulate the cellular activity of the enzyme. With the appreciation of the prevalence and functional relevance of IDRs in proteins, there is a growing interest in the druggability of IDRs and the biophysical methods to address the issue of rational drug design in the absence of hard structural information (35, 36). Indeed, the IDR of a clinically significant nonreceptor PTP, PTP1B, has been successfully shown to be a possible target for inhibitor development (37). As mentioned, the lack of protocols to obtain large amounts of the full-length protein has been a major hurdle toward rational discovery of small molecule regulators of its activity, and we propose that targeting a limited region of the IDR can be explored as an alternative for the development of such agents. As phosphorylation stimulates the catalytic activity, this could open the way for the search for both inhibitors and activators, depending on the biochemical context. Our NMR data show that the IDR proximal region interacts with the catalytic domain *in vitro*. Furthermore, we obtained evidence for a candidate site on the surface of the core domain, about 20 Å from the active site, for the interaction with this region of the IDR (Fig. 5*D*). Biophysical and *in silico* evidence, again from PTP1B, shows that perturbations at sites distal to the active site affect enzyme kinetics *via* long-range allosteric communication pathways that have been proposed as possible routes to exploit for drug discovery (38, 39, 40, 41). The effect of the S325E substitution on the catalytic activity of recombinant PTPN22 *in vitro* is however quite limited and likely unable to alone account for some of the cellular outcomes we observe. This leads us to believe that any such outcome is dependent on the cellular environment by one or more mechanisms, such as altered interactions with potential binding partners or specific substrates, other posttranslational modifications, colocalization, or local concentration effects. The latter would also account for the apparent discrepancy between the kinetics data on one hand and the evidence of an effect of the S325 substitution on the interaction between the interdomain and the catalytic domain in the DXMS experiment on the other. From these considerations, a deeper characterization, beyond the scope of this work, of the allosteric mechanism described here can be called for. Nevertheless, it is reasonable to hypothesize, on the basis of the results presented, that targeting the IDR would constitute a viable strategy to modulate PTPN22 activity.

Another promising aspect of Ser^325^ phosphorylation for future investigation is its dependence on GSK3, a kinase that is actively being pursued as a drug target, though no inhibitors are currently in use for the indications that have been in clinical trials (42). In this respect, multiple reports indicate that TCR signaling would inactivate GSK3 following CD28 costimulation, seemingly at odds with the findings in this work (36, 43, 44). The process of GSK3 inactivation however involves phosphorylation of Ser^9/21^ and the subsequent inhibition of the binding of primed, that is prephosphorylated at the P+4 position, substrates *via* an intramolecular competition mechanism (45). Phosphorylation of substrates that do not have a Ser/Thr residue at P+4, such as PTPN22 (see Fig. S1*B*), is not predicted to be suppressed, which provides an explanation for the apparent contradiction and could even be explored as a possible means for the cell to increase phospho-Ser^325^ levels, as more GSK3 becomes available to modify nonprimed substrates. Further efforts at examining these points will clarify if, and how, targeting GSK3 is a viable strategy to address PTPN22-related pathologies.

Finally, we look at the broader significance of Ser^325^ phosphorylation with a systems biology approach. As a multifunctional regulator of immune cells, PTPN22 is a key player in multiple human autoimmune diseases and response to infection (2, 3). In the signaling pathways profile analyzed following T cell activation by next-generation sequencing, it is noteworthy that the *IL2* gene, which associates with T cell proliferation, was shown to be upregulated in T cells carrying S325E compared to S325A (Fig. 3*E*). PTPN22 gain of function is expected to inhibit IL-2 production (9, 46). However, *IFNG* and other genes involved in cell proliferation were downregulated in S325E cells (data not shown), and IFN-γ has been confirmed to reduce T cell proliferation (47). On the other hand, EGR2 and EGR3, which were confirmed as negative regulators of T cell activation and differentiation and are regulated by antigen and IFN-γ (29, 48), were both upregulated in S325E cells. These observations suggest that Ser^325^ phosphorylation has additional complex systems-level effects on the functioning of T cells. In addition, the link of PTPN22 to genes involved in chromatin remodeling warrants further investigations into open/closed chromatin states using additional targeted assays (*e.g.,* ATAC-seq *etc.*).

A limitation of this work was its use of an imperfect mimic of serine phosphorylation. Glutamate substitution is often used for this purpose; however the single charge introduced in this way only incompletely replicates the chemical effect of the phosphate group. On a related note, we were unable to assess the extent of phosphorylation that occurs in cells, therefore any conclusions obtained from the S325E mutant should not be taken as having a quantitative value when used to predict or interpret the cellular consequences of this signaling event. However, we were encouraged by the observation that the S325E mutation recapitulates well the effects of the phosphorylation in cells and believe that any qualitative conclusions remain valid.

In summary, we have identified a novel site in the interdomain of PTPN22 that is phosphorylated in T cells upon TCR stimulation and have begun to define the biochemical outcomes and signaling events associated with it. Because of the proximity of Ser^325^ to the PTP domain, we have reason to believe that this finding promises to lead to a better understanding of PTPN22 regulation and could create new opportunities for rational discovery of allosteric inhibitors or activators of this medically significant enzyme.

## Experimental procedures

Cells human primary CD4^+^ T cells were isolated and expanded as described before (9). Primary cells and Jurkat E6.1–derived cell lines were cultured in complete RPMI 1640 medium with 10% fetal bovine serum and supplemented with 2 mM L-glutamine, 1 mM sodium pyruvate, 2.5 mg/ml D-glucose, 10 mM Hepes, 100 units/ml penicillin, and 100 μg/ml streptomycin under 5% CO_2_ at 37 °C.

### Primers, plasmids, antibodies, and reagents

All primers used in the study were synthesized by integrated DNA technologies and are summarized in Table 1.

**Table 1:** Primers used in the study

Name	Sequences
pEF3-S325A-F	CAGACTCTTATGCCCCTAATTTACC
pEF3-S325A-R	GGTAAATTAGGGGCATAAGAGTCTG
pEF3-S325E-F	CAGACTCTTATGAGCCTAATTTACC
pEF3-S325E-R	GGTAAATTAGGCTCATAAGAGTCTG
PTPN22 CFLAG-700 (sequencing)	GGACTGGTGTTATTTGTGCT
PTPN22 325-725bp R	TTTTGTCCTTTGTTGGTTCA
S325A AS-F (allele-specific)	CAAGCAGACTCTTATGCC
PTPN22 325-772bp R	CTAAAGTCAAAGGAAGAAGA
S325E AS-F (allele-specific)	GCAAGCAGACTCTTATGAG
HDR807-F	GATGCTGAAATTTCTAAAGGC
HDR807-R	AAAGAGAGCCCAAATCCAAACTT
HDR1183-F	GATCTAATTACTTAGAAGGAG
HDR1183-R	GATTCCAAATAAGAAAAGTTT

HDR, homology-directed repair; PTPN22, protein tyrosine phosphatase nonreceptor type 22.

pEF C-terminal triple FLAG-tagged (3× FLAG) full-length PTPN22 WT plasmid was described before (9). 3× FLAG PTPN22 S325E and S325A plasmids were constructed by site-directed mutagenesis using 3× FLAG PTPN22 WT as template. Plasmids encoding C-terminal 3× FLAG WT and S325E PTPN22_1-330_ and PTPN22_1-340_ were purchased from GenScript. pD1321-AP WT Cas9 plasmid with red fluorescent protein selection marker was purchased from DNA 2.0 (ATUM). NFAT/AP-1 firefly luciferase and *Renilla* luciferase reporters were purchased from Promega. N-terminal His-tagged WT PTPN22_1-330_ and PTPN22_1-340_ were constructed by inserting a stop codon at the appropriate sites in the ORF of PTPN22 (1–419) cloned into the Ncol/XhoI sites of pET28a vector (Novagen) and expressed in *E. coli* BL21(DE3). Mutants were then generated by site-directed mutagenesis. MCS-13× Linker-BioID2-HA vector (19) a gift from Kyle Roux (Addgene plasmid # 80899; http://n2t.net/addgene:80899; RRID:Addgene_80899). All plasmids were confirmed by DNA sequencing.

The polyclonal rabbit anti-human phospho-PTPN22 Ser^325^–specific antibody was custom made by Pacific Immunology. Goat anti-human PTPN22 polyclonal antibody was purchased from R&D systems. Rabbit anti-phospho-Src family (Tyr^416^), anti-LCK, anti-phospho-ZAP70 (Tyr^3^^19^), anti-ZAP70, anti-phospho-PLC-γ Tyr^783^, anti-PLC-γ, and anti-GAPDH antibodies were from Cell Signaling Technology. Mouse anti-FLAG M2 and anti-β Actin mAbs were purchased from Millipore Sigma. Ultra-LEAF purified human CD3 antibody (Clone, OKT3), and Ultra-LEAF purified human CD28 antibody (Clone, CD28.2), brilliant violet (BV421)-conjugated anti-human CD3 antibody, and APC-conjugated anti-human CD69 antibody were purchased from BioLegend. The polyclonal goat anti-mouse Ig used for cross-linking was from BD Biosciences. Horseradish peroxidase–conjugated anti-rabbit IgG, anti-mouse IgG, and anti-goat IgG were purchased from GE Healthcare.

Anti-FLAG M2 magnetic beads, dimethyl sulfoxide (DMSO), G418, β-mercaptoethanol, thrombin, and IPTG were purchased from Millipore Sigma. Pierce protease inhibitor tablets (EDTA-free), Pierce protease and phosphatase inhibitor tablets (EDTA-free), eBioscience Fixable viability Dye eFluor 506 or 780, Dynabeads Human T-Activator CD3/CD28 magnetic beads, DiFMUP, SuperScript III First-Strand Synthesis SuperMix for qRT-PCR, and Platinum PCR SuperMix were purchased from Thermo Fisher Scientific. 2× Laemmli sample buffer was from Bio-Rad. Protein G Sepharose 4 Fast Flow was purchased from GE Healthcare. The dual-luciferase reporter assay system was purchased from Promega. QIAquick Gel Extraction kit, RNeasy Plus Micro Kit, and DNeasy Blood & Tissue kits were purchased from QIAGEN.

### Inhibitors and cell treatment

GSK3-inhibitor IX was purchased from Selleckchem, and SB203580 (p38 MAP kinase inhibitor) was from Cell Signaling Technology. For cell treatments, Jurkat cells were starved for 48 h in basic RPMI 1640 medium and pretreated with the inhibitors for 30 min at 37 °C. Cells were stimulated with or without 1 μg/ml antibodies against human CD3/CD28 and cross-linked with 1 μg/ml rabbit against mouse Ig for the indicated times in the presence of inhibitors or DMSO.

### Generation of homozygous PTPN22 S325A and S325E knock in Jurkat cell lines by CRISPR/Cas9

3× FLAG PTPN22 WT KI Jurkat cells have described before (9). To generate 3× FLAG PTPN22 S325A KI and 3× FLAG PTPN22 S325E KI Jurkat cells, guide RNA targeting PTPN22 genomic DNA nucleotides sequences around Ser^325^ in exon 12 was designed and cloned into pD1321-AP WT Cas9 plasmid. Plasmids containing about 807 bp homology-directed repair (HDR) arms with S325A or S325E mutations were ordered from GenScript. The HDR fragments were amplified by PCR using Platinum PCR SuperMix and purified by QIAquick Gel Extraction kit. The DNA fragments concentration was determined by Nanodrop. For cell lines establishment, 3 × FLAG PTPN22 WT cells were electroporated with 10 μg Cas9 plasmid and 10 μg HDR fragments containing either S325A or S325E mutation. After 24 h, signal cell was sorted into round-bottom 96-well plates by SONY Sorter with red fluorescent protein selection. Cell clones were collected, and genomic DNA was extracted by DNeasy Blood & Tissue kits. Positive clones were screened by PCR amplification using allelic-specific primers (Table 1). RNA was extracted from positive candidate clones by RNeasy Plus Micro Kit, and complementary DNA was synthesized by SuperScript III First-Strand Synthesis SuperMix. The mRNA mutations were further confirmed by DNA sequencing.

### BioID2-PTPN22 Jurkat cell line construction

A full-length PTPN22 ORF was cloned into MCS-13× Linker-BioID2-HA vector and confirmed by DNA sequencing. The recombinant vector was electroporated into PTPN22 KO Jurkat cells. BioID2-HA empty vector electroporated cells served as a control. The cells were treated with 0.5 mg/ml G418 after 24 h for 7 days. Next, cells were washed twice with PBS and stained with Viability Dye eFluor 780 for 30 min at 4 °C. Single cell was sorted into 96-well plates as described above and cultured with 0.5 mg/ml G418 for 2 weeks. Cell clones were collected, and genomic DNA was extracted. Positive clones were screened by PCR amplification and DNA sequencing using PTPN22 primers (Table 1). PTPN22 expression was confirmed by Western blot analysis.

### IP, SDS-PAGE, and Western blot

For IP, cells were collected and washed twice with cold PBS, and were lysed in 1× TNE lysis buffer (50 mM Tris-Base, pH 7.4, 150 mM NaCl, 1 mM EDTA) supplied with proteinase inhibitors and 1 mM PMSF and incubated on ice for 15 min. The lysates were centrifuged at 15,000 rpm/min for 10 min at 4 °C to remove the cell debris. The supernatants were harvested and used for IP using the indicated antibodies or magnetic beads bound with specific antibodies as described before (9). The immunoprecipitated proteins were dissociated from antibodies-bound beads by adding 2 × Laemmli sample buffer containing 5% β-mercaptoethanol and boiled for 5 min. Next, the proteins were separated by SDS-PAGE, and transferred to a nitrocellulose membrane using a semidry transfer system. Interesting proteins were probed with specific antibodies and then visualized through an enhanced chemiluminescence detection system. For recombinant protein gel electrophoresis, fractions were diluted with 2 × Laemmli sample buffer containing 10% β-mercaptoethanol, separated by Tris-glycine eXtended stain-free gel (Bio-Rad) and visualized by stain-free imaging under UV light.

### Recombinant protein expression and purification

*E. coli* BL21 (DE3)-expressing plasmids encoding for recombinant proteins were grown at 37 °C and induced with 0.1 mM IPTG at 18 °C for 18 h. For WT and mutant PTPN22_1-330_ and PTPN22_1-340_ recombinant proteins purification, cell pellets were lysed by freeze thawing in the presence of 200 μg/ml chicken egg lysozyme (Sigma) and treated with 10 μg/ml DNaseI (Roche) for 1 h on ice. Supernatants were cleared by centrifugation at 4 °C for 1 h at 15,000 rcf. Target proteins were purified by nickel-nitrilotriacetic acid affinity chromatography (QIAGEN) and desalting (Bio-Scale Mini Bio-Gel P-6 Desalting Cartridge, Bio-Rad). The N-terminal His-tag was then cleaved by thrombin digestion at room temperature for 1 h and stopped with 1 mM benzamidine. Digested products were concentrated and purified by size-exclusion chromatography (Superdex 200, Cytiva Lifescience). All purified proteins were confirmed by stain free SDS-PAGE.

### Dual-luciferase reporter assay

The dual-luciferase reporter assay was performed as previously described (9). Briefly, PTPN22 KO Jurkat cells were electroporated with control vector, 3× FLAG PTPN22 full-length WT, S325E, S325A or the truncation (PTPN22_1-330_ WT and variants, PTPN22_1-340_ WT and variants) plasmids along with NFAT/AP-1 firefly and *Renilla* luciferase reporter plasmids, respectively. Next, cells were stimulated with 1 μg/ml antibodies against human CD3 and CD28 for 6 h. Then, cells were harvested, and dual-luciferase reporter assays were performed. PTPN22 expression was confirmed by Western blotting.

### Flow cytometry

For CD69 and CD3 expression, 3× FLAG PTPN22 WT, S325A, and S325E KI Jurkat cells were starved in basic RPMI 1640 medium for 48 h. Cells were stimulated with or without (Mock) 1 μg/ml antibodies against human CD3 and CD28 for 4 h. Cells were harvested and washed twice with FACS buffer (2.5% fetal bovine serum, 1 mM EDTA) and blocked with human Fc blocker (BD Biosciences) for 15 min at room temperature. After staining with Viability Dye eFluor 506, APC-conjugated anti-human CD69 antibody, and BV421-conjugated anti-human CD3 antibody at room temperature for 30 min, cells were washed three times with FACS buffer and assessed by a ZE5 flow cytometer (Bio-Rad). Results were analyzed by FlowJo software (Treestar, https://www.flowjo.com/solutions/flowjo).

### *In vitro* phosphatase activity assays

3× FLAG-tagged PTPN22 was immunoprecipitated by anti-FLAG M2 magnetic beads from lysates of PTPN22 KO Jurkat cells overexpressing full-length WT, S325E, or S325A PTPN22. Beads binding with target proteins were resuspended in kinetics buffer (50 mM Tris, pH 7.2, 1mM DTT, 0.01% Triton X-100), and combined with 1 mM DiFMUP dissolved in kinetics buffer containing 8% DMSO.

Recombinant proteins were purified as described above and diluted to a concentration of 1 nM in kinetics buffer. DiFMUP was serially diluted (0, 8, 20, 40, 80, 200, 400, and 800 μM) in kinetics buffer containing 8% DMSO. Diluted proteins and DiFMUP were mixed (1:1) in round-bottom black 96-well polystyrene microplates (Corning) with three replicates for each condition. Phosphatase activity was then assessed by monitoring fluorescence signals on a Tecan Infinite M1000 plate reader at wavelengths of 358 nm (excitation) and 455 nm (emission) at 37 °C for 30 min as performed previously (49). Immunoprecipitated PTPN22 from PTPN22 KO Jurkat cells was detected by Western blotting.

### Phospho-MS and bulk RNA sequencing

All the Jurkat cell lines were starved for 48 h and then stimulated for different purposes. For PTPN22 phosphorylation sites mapping, 3× FLAG PTPN22 KO or WT Jurkat cells were stimulated with 1 μg/ml antibodies against human CD3 and CD28 for 5 min and then harvested, washed twice with ice-cold PBS, lysed in 1× TNE lysis buffer supplied with proteinase inhibitors and 1 mM PMSF. The lysates were processed as described above and PTPN22 protein was affinity-purified using anti-FLAG M2 magnetic beads (Sigma). The immunoprecipitated proteins were dissociated from beads by adding 50 mM Tris-Base, pH 7.4, 150 mM NaCl containing 1 mg/ml FLAG peptides and incubated at 4 °C for 1 h with rotation. The eluted proteins were collected and confirmed by protein gel electrophoresis and Coomassie blue staining. For global phospho-MS, 3× FLAG PTPN22 WT, S325A, and S325E KI Jurkat cells were stimulated with or without (Mock) 1 μg/ml antibodies against human CD3 and CD28 for 5 min. Cells were harvested, washed twice with ice-cold PBS, and kept at −80 °C for further processing. For bulk RNA-seq, 3× FLAG PTPN22 WT, S325A, and S325E KI Jurkat cells were stimulated in 12-well plates with or without (Mock) 1 μg/ml antibodies against human CD3 and CD28 for 16 h. Cells were collected and washed twice with ice-cold PBS and lysed in into Trizol-LS and stored in −80 °C until further processing.

### Preparation of samples for proteomics analysis

Cell pellets processed for MS were first dried down to remove liquid and then resuspended in 1 ml lysis buffer (6 M urea, 7% SDS, 50 mM Tris, pH 7.1), vortexed for 20 min at room temperature, and sonicated for three rounds (10 on, 10 off, 20% peak intensity). Samples were then treated with 5 mM DTT at 47 °C for 30 min, cooled to room temperature, and alkylated using 30 μl 500 mM iodoacetamide in the dark for 45 min, followed by quenching with 10 μl 500 mM DTT. To each reduced and alkylated sample, 100 μl 12% phosphoric acid was added, followed by 7:1 vol/vol of binding buffer (90% MeOH, 10% 1 M triethylammonium bicarbonate). Each sample was then passed through an “S-Trap” column (Protifi, #C02-96-well-1) to capture proteins and washed five times with binding buffer. To each sample, a total of 50 μg trypsin resuspended in in 50 mM triethylammonium bicarbonate was added and digestion was carried out for 3 h at 47 °C. Peptides were then eluted and dried down. Each sample was then desalted, dried down, and then quantified (Thermo PepQuant). Fifty micrograms of each sample was taken for proteomic analysis and 2.5 mg was taken for further phosphoenrichment and all were dried down. Phosphoenrichment sample sets were resuspended 4:1 with washed Titansphere TiO, 5 μm beads (GL Sciences Inc, CAT# 502075000) in 1.8 ml of binding buffer (2 M lactic acid) and placed on rotator for 1 h. After incubation, bead-peptide mix was washed (50% acetonitrile, 0.1% TFA) to remove unbound fractions, and eluted into fresh tube twice with elution buffer (50 mM KH_2_PO_4_). The eluted peptides were dried down, desalted, and dried down again to tandem mass tag (TMT) labeling.

Both proteome and phosphoenriched sample sets were labeled with TMT-16 (Thermo Fisher Scientific). Briefly, desalted and dried samples were resuspended in 50 μl of 30% acetonitrile, 20% 1 M Hepes and vortexed for 20 min. After spinning samples down, each sample received 7 μl of a single TMT channel (1, 2, 3, 4, 5, 6, 7, 8, 9, 10, 11, 12, 13, 14, 15, 16) and mixed thoroughly by pipette. Each sample was incubated for 1 h and the reaction was stopped by adding 8 μl 5% hydroxylamine. Finally, 50 μl 1% TFA was added to each channel, after which all channels related to the specific subset were mixed and dried down. These samples were then once again desalted and dried down.

Each TMT-16 plex experiment was then resuspended in 110 μl 25 mM ammonium bicarbonate and fractionated using high-pH reverse phase fractionation on an offline HPLC (Thermo Fisher Scientific Ultimate 3000, BioBasic c18 column) into 12 fractions over the course of a 78-min increasing organic gradient (starting at 3 min [10% for proteome, and 3.5% for phosphor fraction], and ending at 60 min [40% for proteome, 28% for phospho fractions]). These fractions were then dried down and resuspended in 20 μl 5% formic acid and 5% acetonitrile in glass vials for further MS analysis.

### MS-based data acquisition

Samples were loaded into a Thermo Scientific Easy-nLC 1000 coupled to an Acclaim PepMap c18 column and an EasySpray NG (Thermo Fisher Scientific). Mass spectrometer acquisition methods are detailed in Lapek *et al.* (50). Briefly, samples loaded onto column were subjected to increasing percent organic solvent over the course of 180 min gradient method. TMT-labeled were fragmented in MS3 with synchronous precursor selection enabled. Data was acquired using a Thermo Scientific Orbitrap Fusion.

### MS data processing and analyses

All data was searched using Proteome Discoverer 2.5 (https://knowledge1.thermofisher.com/Software_and_Downloads/Chromatography_and_Mass_Spectrometry_Software/Proteome_Discoverer/Proteome_Discoverer_Operator_Manuals/Proteome_Discoverer_2.5_overview). Each fraction set was searched against a reference human database downloaded from UniProt (uniprot.org) on 5/16/2022. Fixed modifications included carbamidomethylation (+57.02146 Da) and TMTpro (+304.2071 Da). Variable modifications included oxidation (+15.9949 Da) and STY phosphorylation (+79.9663 Da). All peptide-spectrum matches and proteins were filtered *via* combined false discovery rate of 0.01 using a target-decoy strategy to generate underlying score distributions.

Searched proteins were normalized to most intense channel (done internally by Proteome Discoverer). Data was then further Log_2_-transformed. Only features with a full suite of values present were used in downstream analysis. All nonphosphopeptides present in the phosphoenrichment sample set were removed. Each replicate was compared separately to reduce batch bias. Stimulated S325E protein/phosphopeptide abundances were compared to that of WT conditions (D_*stim_i*_ = S325E - WT), and equivalent mock stimulation conditions was used to control for background (D_*mock_i*_ = S325E - WT). To account for background effects present in mock stimulated conditions, we subsequently calculated Δ between D_*stim_i*_ and D_*mock_i*_ to generate a final “enrichment” delta between mock and stimulated conditions. The same analyses were undertaken for alanine mutant pairs. Results of both replicates were then compared and subjected to a consensus factor of >0.4 residual delta was required to be passed into any further analysis.

Enriched proteins/peptides passing the consensus analysis were subjected STRING-DB protein–protein interaction networks to determine any underlying changes in ontology terms. Resulting network was further subsetted using Markov clustering with an inflation parameter of 1.4 to 1.6. The resulting network with the highest number of features was plotted. The highest scoring feature (network strength) with significant enrichment score is reported.

### Bulk RNA-seq

mRNA sequencing was completed at La Jolla Institute for Immunology Next-Generation Sequencing Core Facility (RRID:SCR_023107). Briefly, RNA samples were extracted by using RNeasy Micro kit (Qiagen), and a library was prepared with NEBNext Ultra II Directional RNA library prep kit for Illumina. Sequencing was performed on a NovaSeq 6000 (S10OD025052) at 25 M reads/sample using 2 × 50 bp paired-end sequencing. The quality of data was assessed using the FastQC software (https://www.bioinformatics.babraham.ac.uk/projects/fastqc). Sequences were aligned to the Genome Reference Consortium Human Build 38 (GRCh38) reference genome using STAR version 2.5.4b (51). Genes with less ten counts were excluded from subsequent analysis. DEGs were determined using DESeq2 (52) version 1.36.0 using a ± 2-fold threshold and false discovery rate-adjusted *p* value <0.05.

### NMR spectroscopy

#### Protein expression and purification

Human PTPN22 DNA (amino acids 1–360) was synthesized by Integrated DNA Technologies, Inc. and subcloned into pRP1B as previously described (53). The expression plasmids of PTPN22 catalytic domain (residues 1–299) and the PTPN22 interdomain (residues 299–360) were amplified using PCR and inserted into pRP1B and pTHMT, respectively. The PTPN22 interdomain variant S325E was generated using site-directed mutagenesis (QuickChange; Agilent). The purification of PTPN22 1 to 299 and 299 to 360 were carried out as follows: *E. coli* BL21(DE3) cells (Agilent) expressing PTPN22 were resuspended in lysis buffer (50 mM Tris pH 8.0, 50 mM NaCl, 0.5 mM tris(2-carboxyethyl)phosphine, 0.5% Triton X-100), lysed by high-pressure homogenization (Avastin C3), and then centrifuged (45,000*g*, 45 min, 4 °C). The supernatant was then loaded onto a HisTrap HP column (Cytiva) pre-equilibrated with buffer A (50 mM Tris pH 8.0, 500 mM NaCl, 0.5 mM tris(2-carboxyethyl)phosphine, 5 mM Imidazole), and bound proteins were eluted using a 5 to 300 mM imidazole gradient. Fractions containing PTPN22 were pooled, dialyzed into buffer A, and incubated with Tobacco Etch Virus protease. After 16 h at 4 °C, the cleavage reaction was loaded onto a nickel-nitrilotriacetic acid column (Invitrogen) and the cleaved PTPN22 protein was isolated in the flow-through. For the PTPN22 interdomain peptides, an additional heat purification step, incubation at 80 °C for 10 min, was also performed. All proteins were purified in a final step using size-exclusion chromatography (Superdex 75 26/60 [Cytiva]) equilibrated with 10 mM Bis-Tris pH 5.6, 100 mM NaCl (1.0 M NaCl for the S325E mutant peptide), 5 mM DTT.

#### NMR spectroscopy

All NMR data were collected on a Bruker Ascend NEO 600 MHz spectrometer equipped with a TCI HCN z-gradient cryoprobe at 283 K. NMR spectra for the assignment of PTPN22 299 to 360 were acquired using (^13^C,^15^N)-labeled protein at a final concentration of 0.3 mM in 10 mM Bis-Tris pH 5.3, 100 mM NaCl, 5 mM DTT, and 90% H_2_O/10% D_2_O. The following spectra were used to complete the sequence specific backbone assignments: 2D [^1^H,^15^N] HSQC, 3D HNCA, 3D HN(CO)CA, 3D HNCACB, 3D CBCA(CO)NH, 3D (HA)CANCO, and 3D HNCO. All spectra were processed with Topspin 4.1.3 (Bruker Billerica), and Cara (http://cara.nmr.ch) was used for the sequence-specific chemical shift assignments.

The interaction between the PTPN22 interdomain or its variant S325E with the catalytic domain 1 to 299 was studied by direct comparison of the 2D [^1^H, ^15^N] HSQC spectra of free (^15^N)-labeled PTPN22 299 to 360 with and without a 12-fold molar excess of the PTPN22 catalytic domain. The spectra were processed using NMRPipe (54) and analyzed with POKY (https://poky.clas.ucdenver.edu).

### Deuterium exchange mass spectrometry

WT PTPN22 was used to optimize the quenching conditions for hydrogen/DX experiments as previously described (55) using an immobilized pepsin column (16 μl bed volume). The coverage maps of identified peptides were compared, and 0.8 M GuHCl/80 mM tris(2-carboxyethyl)phosphine quench buffer was selected for the exchange experiments. Coverage of the polypeptide sequence was 100%.

All exchange stock solutions of PTPN22 (WT and S325E) were made to contain 1.2 mg/ml of protein, 8.3 mM Tris, 150 mM NaCl, pH 7.2 and kept on ice. Exchange experiments were initiated by adding 66 μl of exchange stock solutions to 198 μl of D_2_O containing buffer (8.3 mM Tris, 150 mM NaCl, pDread 7.2) and incubating for various times (10, 100, 1000, 10,000, and 100,000 s) at 0 °C. At each indicated time, 16 μl of exchange reaction solution was taken out, mixed with 24 μl of ice-cold quench buffer, and frozen on dry ice after incubating on ice for 5 min. Nondeuterated and equilibrium deuterated control samples were also prepared for back exchange correction. All frozen samples were thawed out at 4 °C and subjected to proteolysis and LC/MS analysis. All the columns were kept at 0 °C to minimize back exchange. The deuterium incorporation of deuterated peptides was determined using HDXaminer (Sierra Analytics, LLC), which calculates centroid values of each peptide. Back-exchange corrections were calculated as previously described (56). Ribbon Maps were generated with an in-house Excel macro and MatLab scripts.

### Statistical analysis

Protein expression and phosphorylation were assessed by Western blotting and were quantified by Image J software (https://imagej.net/ij). Parametric tests were used on normally distributed variables as assessed by the Shapiro–Wilk test. Statistical analyses were performed with GraphPad Prism 9.2.0 software (https://www.graphpad.com/updates) by using one- and two-way ANOVA, Kruskal–Wallis test, Kolmogorov–Smirnov test, and Mann–Whitney test as indicated in the Figure legends. Error bars represent mean ± SEM and are from at least three biological repeats. Significant differences were defined as ∗*p* <0.05, ∗∗*p* < 0.01, and ∗∗∗*p* < 0.001.

## Data availability

All data are contained within the manuscript.

The MS proteomics data have been deposited in PRIDE/ProteomeXchange with the accession numbers PXD048062 and PXD048064.

The Bulk RNA-seq data has been deposited in Gene Expression Omnibus with assigned Gene Expression Omnibus accession number GSE264373.

The NMR data generated in this study have been deposited in the BioMagResBank database under accession code BMRB 52262.

## Supporting information

This article contains supporting information (58).

## Conflict of interest

The authors declare that they have no conflicts of interest with the contents of this article.
