# Supplementary material for: A novel gain-of-function phosphorylation site modulates PTPN22 inhibition of TCR signaling
Source: J Biol Chem. 2024 May 21;300(6):107393. doi: 10.1016/j.jbc.2024.107393 (PMC11237943; doi:10.1016/j.jbc.2024.107393)
Supplement: Supporting Figures [file mmc1.docx]

**\**

**
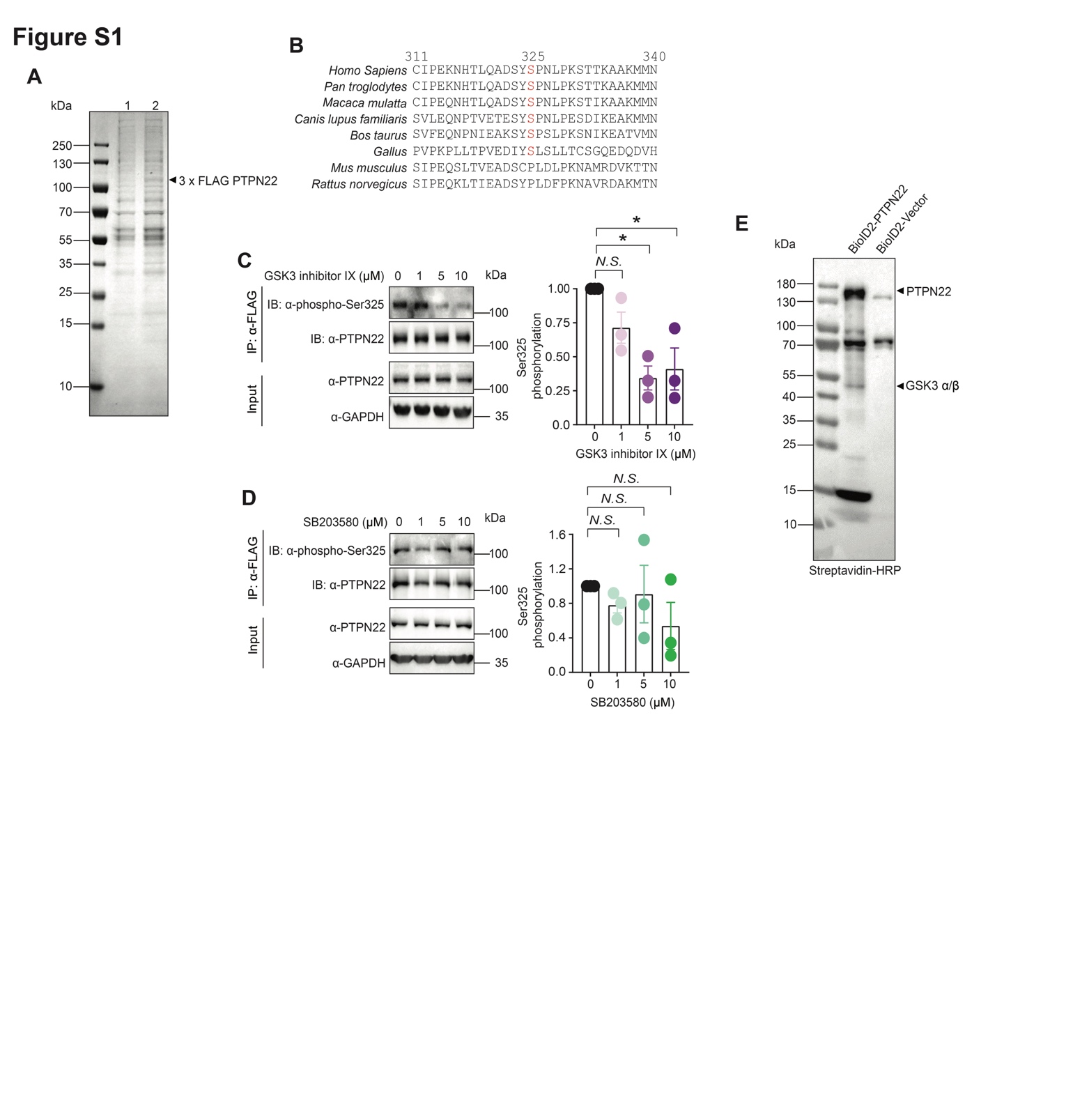
**

**Fig. S1. PTPN22 purification and effects of inhibitors on PTPN22 Ser^325^ phosphorylation.**

(**A**) Coomassie stained SDS-PAGE of 3× FLAG PTPN22 immunoprecipitated from PTPN22 KO (Lane 1) or 3× FLAG tagged WT PTPN22 Jurkat cells (Lane 2) cross-linked with antibodies against human CD3/CD28 and eluted with a FLAG peptide. Data is representative of three independent biological replicates. (**B**) Multiple sequence alignment of amino acid sequences around PTPN22 Ser^325^ (red) across various species. (**C** and **D**) Immunoprecipitation analysis of phospho-Ser^325^ in lysates of 3× FLAG WT PTPN22 KI Jurkat cells treated with the indicated concentrations of GSK3 inhibitor IX (**C**) or p38 MAPK inhibitor (SB203580) (**D**). Western blot analyses shown are representative of three independent experiments for each inhibitor (left panel). Quantification of phosphorylated Ser^325^ normalized to relative total PTPN22 protein from three independent experiments (right panels). Statistical significance was assessed by using the Kruskal-Wallis test, **P*<0.05, *N.S.*, not significant. (**E**) Western blot analysis of biotinylated proteins from lysates of BioID2-HA empty vector or BioID2-WT PTPN22 Jurkat cells using HRP conjugated streptavidin. Panel shows a representative blot from three independent experiments.

**
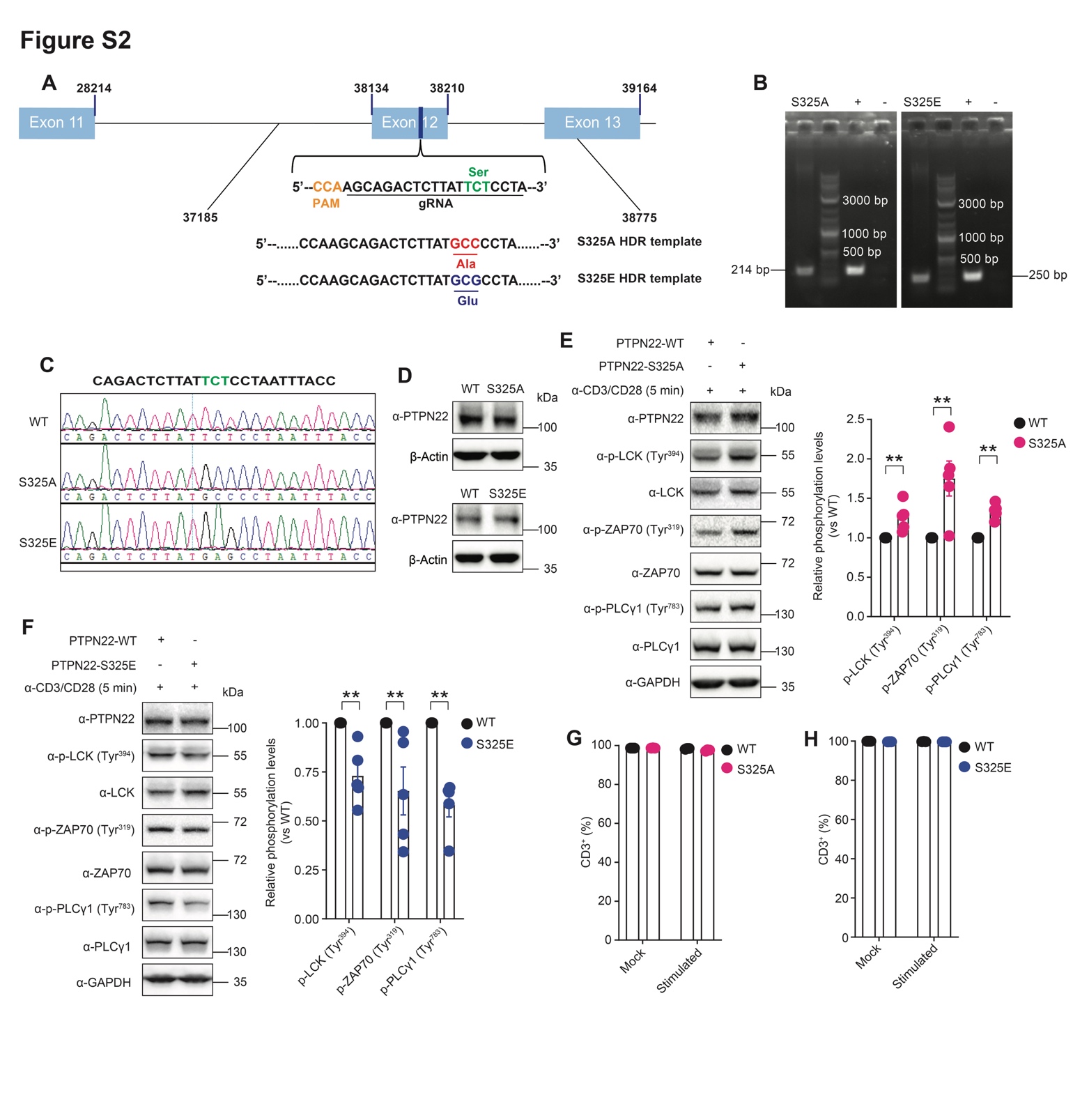
**

**Fig. S2. Generation of 3× FLAG PTPN22 S325A and S325E KI Jurkat cell lines and TCR signaling pathway in PTPN22 KO cells overexpressing the mutants.**

(**A**) Schematic of CRISPR/Cas9 mediated PTPN22 S325A and S325E KI cell line construction. (**B**) Agarose gel electrophoresis to identify PCR products amplified from PTPN22 KI Jurkat cells using allele-specific primers. The genomic DNA isolated from S325A or S325E single cell colonies served as templates. +, positive control; -, negative control. The target PCR amplification fragments were indicated. (**C**) cDNA sequencing results from PTPN22 WT, S325A or S325E KI Jurkat cell lines. (**D**) Western blot analysis of PTPN22 expression in 3× FLAG PTPN22 WT, S325A or S325E KI Jurkat cell lysates. (**E** and **F**) TCR signaling analysis in PTPN22 KO cells overexpressing 3× FLAG PTPN22 WT, S325A or S325E. Cells were treated with antibodies against human CD3/CD28 and cross-linked with rabbit anti-mouse Ig antibody for 5 minutes. Western blot analysis of the phosphorylation levels of LCK (Tyr^394^), ZAP70 (Tyr^319^), and PLC-γ (Tyr^783^) in cells overexpressing 3× FLAG PTPN22 WT and S325A mutant (**E**) or 3× FLAG PTPN22 WT and S325E mutant (**F**). Left panels show representative Western blots from five independent experiments. Histograms show quantification of phosphorylated LCK (Tyr^394^), ZAP70 (Tyr^319^), and PLC-γ (Tyr^783^) normalized to total protein (Right panels). Statistical significance was assessed using the Kolmogorov-Smirnov test, ** *P*<0.01. (**G** and **H**) Flow cytometry analysis of CD3 expression in 3× FLAG PTPN22 WT and S325A (**G**) or S325E (**H**) KI Jurkat cells treated with (Stimulated) or without (Mock) antibodies against human CD3/CD28. Dot plots show percentages of CD3^+^ positive cells from three independent experiments.


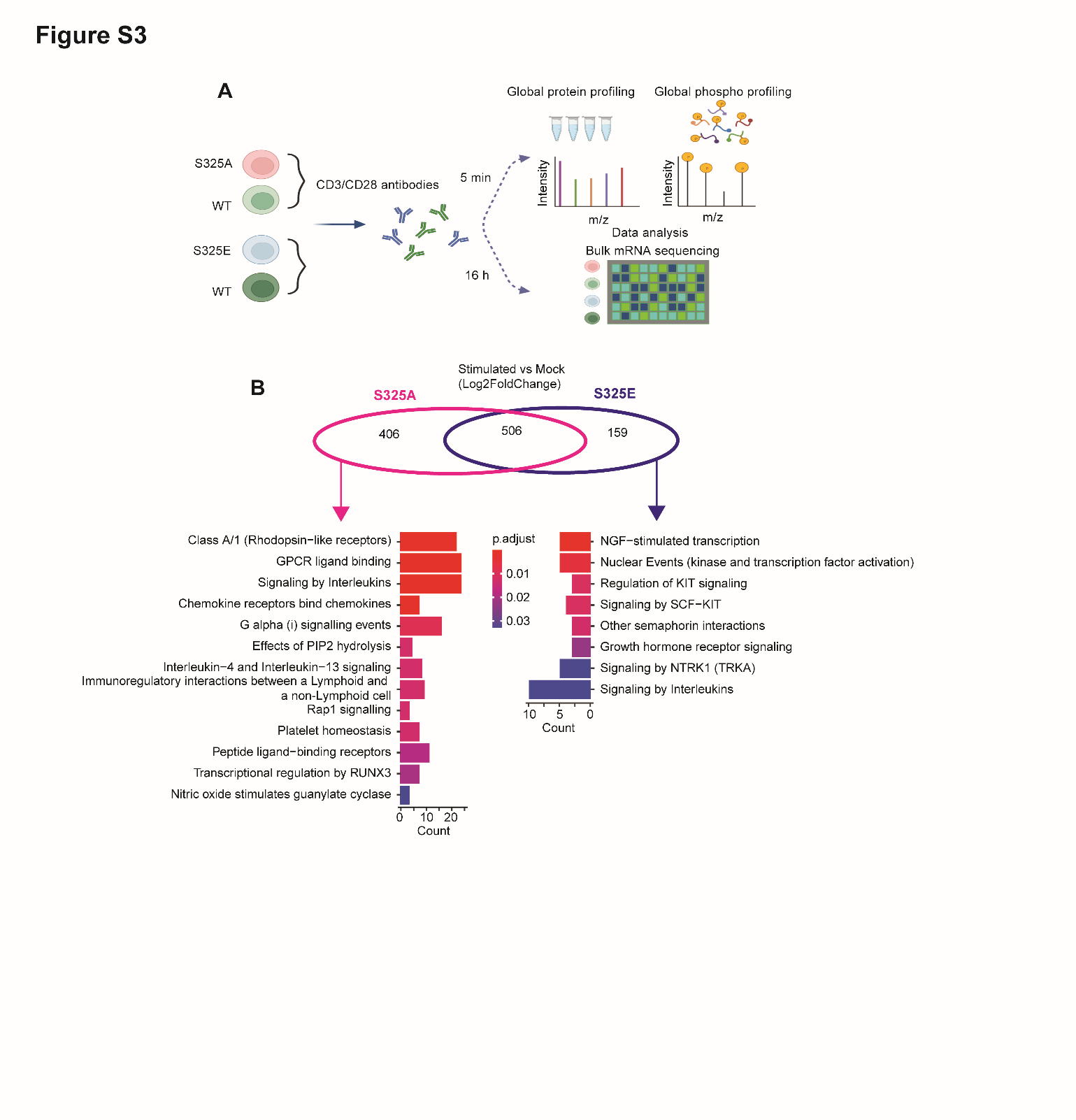


**Fig. S3. Global phosphoproteomics and bulk RNA sequencing.**

(**A**) Diagrammatic sketch of S325A, S325E and their parallel WT Jurkat cells used for mass spectrometry-based proteomics and transcriptomics. (**B**) Reactome pathway analysis of unique differentially expressed genes (406 in S325A group and 159 in S325E group, ≥ 2-fold and adjusted p-value < 0.05: Stimulated vs Mock). The length of the bars represents gene numbers in each group.


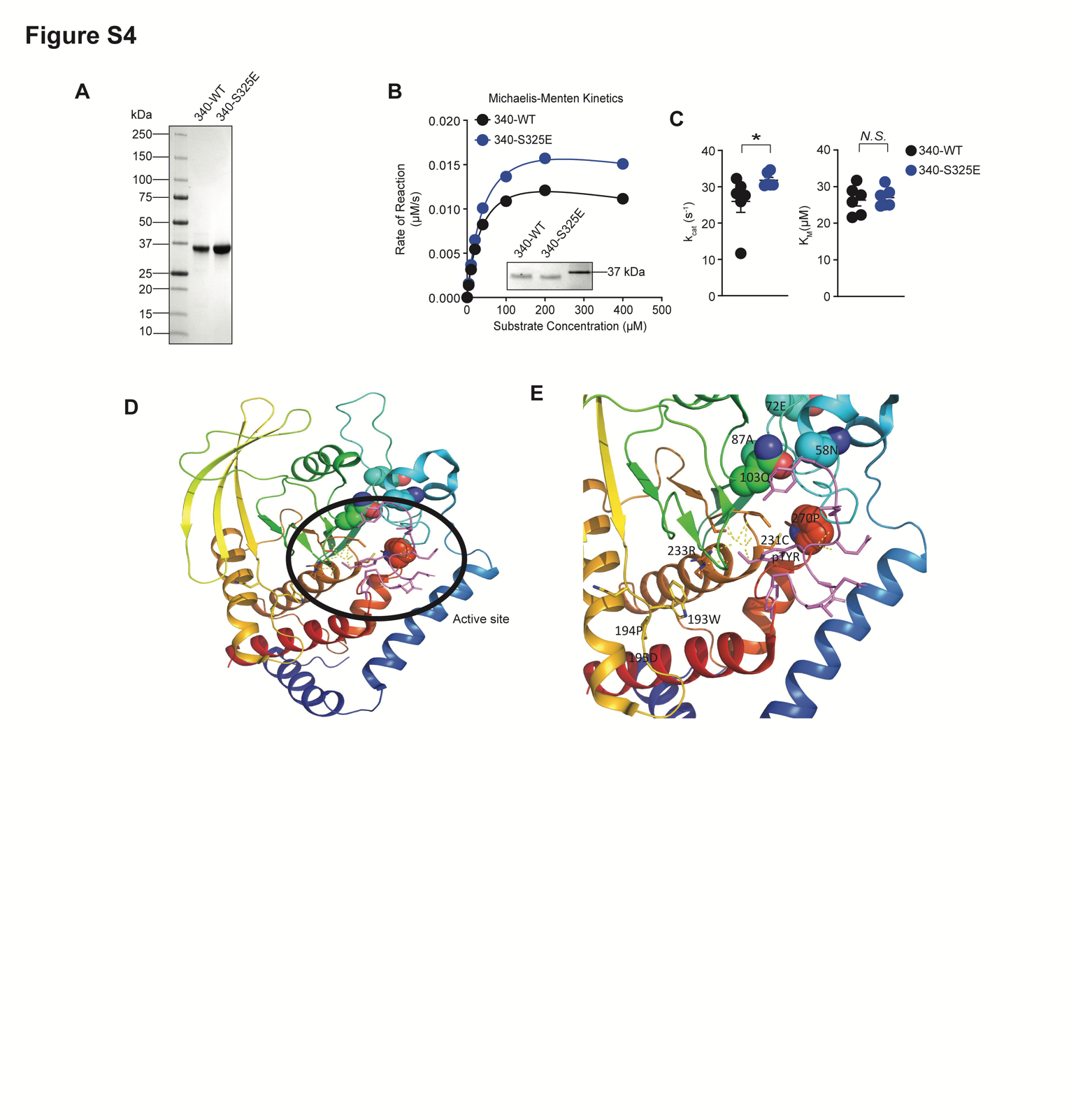


**Fig. S4. Purification of recombinant proteins and structural models.**

(**A)** Representative SDS-PAGE of final purified samples of PTPN22_1-340_ WT and S325E recombinant proteins**. (B** and **C**) Phosphatase activity assays were performed using recombinant WT or S325E PTPN22_1-340_ and DiFMUP as a substrate. (B) Representative Michaelis-Menten curve of five independent experiments each with three technical replicates. Representative SDS-PAGE of 1 µM PTPN22_1-340_ WT and S325E purified proteins is shown to confirm that the same amount of protein was used for all mutants. (C) Dot plot shows *k_cat_ and K_M_*, each data point represents one of five individual experiments performed as in (B). Statistical significance was assessed using the Mann-Whitney test, ** *P*<0.01, *N.S.*, not significant. (**D**) Ribbon representation and (**E**) close-up of the active site and surrounding residues in the structure of PTPN22 catalytic domain (blue to red) with a consensus substrate phosphopeptide bound (purple, PDB code 3OLR). The major residues directly involved in catalysis are highlighted and those in areas affected in the DX/MS experiment are shown as spheres. Yellow dash lines represent hydrogen bonds engaging phosphotyrosine at the catalytic site. The picture was produced using PyMol ([58](#_ENREF_58)).
